# No effect of rifaximin on soluble CD163, mannose receptor or type III and IV neoepitope collagen markers in decompensated cirrhosis: Results from a randomized, placebo controlled trial

**DOI:** 10.1371/journal.pone.0203200

**Published:** 2018-09-05

**Authors:** Nina Kimer, Natasja Stæhr Gudmann, Julie Steen Pedersen, Søren Møller, Mette Juul Nielsen, Diana Julie Leeming, Morten Asser Karsdal, Holger Jon Møller, Flemming Bendtsen, Henning Grønbæk

**Affiliations:** 1 Gastro Unit, Medical Division, Copenhagen University Hospital Amager Hvidovre, Hvidovre, Denmark; 2 Centre of Diagnostic Imaging and Research, Department of Clinical Physiology and Nuclear Medicine, Copenhagen University Hospital Hvidovre, Hvidovre, Denmark; 3 Nordic Bioscience, Fibrosis Biology and Biomarkers, Herlev, Denmark; 4 Department of Clinical Biochemistry, Aarhus University Hospital, Aarhus, Denmark; 5 Department of Hepatology & Gastroenterology, Aarhus University Hospital, Aarhus, Denmark; Kaohsiung Medical University, TAIWAN

## Abstract

**Background and aims:**

Macrophages play a significant role in chronic liver disease as reflected by elevated soluble (s)CD163 and mannose receptor (sMR) levels and associated with liver disease severity and prognosis. Extracellular matrix remodelling associated with fibrogenesis may be affected by systemic inflammation induced by bacterial translocation. Therefore, we aimed to investigate the effect of rifaximin-α, an antibiotic with effect on gut bacteria, on sCD163, sMR, and collagen metabolites.

**Methods:**

Fifty-four clinically stable patients with decompensated cirrhosis were randomized to 4 weeks treatment with rifaximin-α (n = 36) or placebo (n = 18). Macrophage markers sCD163, sMR and markers of collagen fibrogenesis (C3M and C4M) and formation (PRO-C3 and P4NPS7) were analysed in plasma before and after treatment.

**Results:**

sCD163 and sMR levels were associated with liver disease severity (MELD score, sCD163 rho = 0.47, p<0.001 and sMR rho = 0.37, p = 0.005). There was no effect of Rifaximin-α on sCD163 levels (median (range) sCD163 5.64(2.02 to 10.8) at baseline versus 4.42(1.98 to 8.92) at follow-up in the rifaximin-α group and 4.85 (2.29 to 12.1) at baseline versus 4.32 (1.98 to 12.4) at follow-up in the placebo-group), p = 0.34); nor sMR levels, p = 0.34.

Also in patients with elevated lipopolysaccharide binding protein (> 5.9 μg/ml, 38 patients) there was no effect of rifaximin-α on sCD163 (p = 0.49) or sMR levels (p = 0.32).

**Conclusion:**

We confirmed that macrophage activation markers sCD163 and sMR are directly associated to liver disease severity (MELD score). However, rifaximin-α has no effect on sCD163, sMR or collagen markers in decompensated cirrhosis and does therefore not seem to interfere with macrophage activation or fibrogenesis.

## Introduction

Portal hypertension (PH) in liver cirrhosis imposes a high risk of complications and increased mortality[1]. PH leads to oesophageal varices and risk of variceal bleeding, ascites formation and systemic vascular dysfunction, including peripheral vasodilation and a hyperdynamic circulation, which is involved in the hepatorenal syndrome and renal failure in cirrhosis [2, 3]. Several factors contribute to the development of PH. Formation of fibrosis in the liver parenchyma causes a mechanic disruption of the liver architecture, and endothelial dysfunction activates contractile properties in the hepatic stellate cell [4, 5]. Also, an increased inflammatory activity in cirrhosis adds to upregulation of innate immune cells and macrophage activation [6, 7]. The degree of macrophage activation can be determined by soluble (s) CD163 and soluble mannose receptor (sMR), which are shed from activated macrophages in the liver [8, 9]. Levels of sCD163 are related to the degree of PH, risk of variceal bleeding, and may be used as a prognostic marker in cirrhosis [9–11].

In liver fibrosis, extensive remodelling of the extracellular matrix such as the fibrillar type III collagens from the interstitial matrix is ongoing [12]. Type III collagen formation and degradation can be evaluated by the serological markers PRO-C3 and C3M respectively [13, 14]. Especially PRO-C3 is found to be associated with fibrosis stage and prognostic for progression of fibrosis [15, 16]. Type IV collagen is primarily found in the basement membrane and it is also subject to extensive remodelling in liver fibrosis. Changes in type IV collagen can be measured by the markers P4NPS7 and C4M being elevated in advanced fibrosis at least in hepatitis B and C [17]. These biomarkers may also be associated with PH, and has the potential to be non-invasive markers of portal hypertension [18]. Markers of fibrogenesis may also be affected by systemic inflammation induced by bacterial translocation [19]. Associations between sCD163 and fibrosis have been demonstrated in patients with cirrhosis due to viral hepatitis [20].

Rifaximin-α is a non-absorbable antibiotic used in the prevention of hepatic encephalopathy [21]. Due to its broad spectrum and effect on gut bacteria, studies have investigated its impact on gut bacteria and possible effects in prevention of bacterial translocation from the gut and thereby prevention of systemic inflammation [22, 23]. The pathophysiological mechanisms of rifaximin-α are still scarcely elucidated.

In this randomized trial (NCT01769040) we aimed to investigate the relations between intestinal decontamination with rifaximin-α and the markers of macrophage activation sCD163 and sMR, in addition to selected neoepitope markers reflecting type III (PRO-C3 and C3M) and IV (P4NPS7, C4M) collagen remodelling in patients with advanced liver cirrhosis. We hypothesized that rifaximin-α treatment would ameliorate both inflammatory and fibrosis markers.

## Materials and methods

### Patients

In a randomized, placebo controlled and double blind clinical trial fifty-four stable patients with liver cirrhosis and ascites were randomized to rifaximin-α (XIFAXAN, Norgine Denmark A/S, for Alfasigma S.p.A., Bologna, Italy) or placebo for 28 days.

Inclusion criteria were diagnosis of cirrhosis verified by clinical, biochemical, and ultrasound findings; clinical or ultrasound-verified ascites within the last three months; age 18 to 80 years; and portal hypertension with a hepatic venous pressure gradient ≥ 10 mmHg. Exclusion criteria were cardiac or respiratory failure, invasive cancer within the past five years, clinical or biochemical signs of infection, antibiotic treatment 14 days prior to inclusion, overt hepatic encephalopathy, kidney failure with serum creatinine above 200 μmol/l, transfusion-requiring bleeding within one week prior to inclusion, blood hemoglobin level below 5.5 mmol/l, continuous abuse of alcohol with symptoms of withdrawal; or expected survival of less than 3 months. In- and exclusion criteria, as well as basal characteristics of the subjects have been described previously [23, 24]. Patients were invasively characterized with liver vein catheterization and measurement of cardiac output and hepatic venous pressure gradient, along with glomerular filtration rate, assessment of hepatic encephalopathy and biochemistry. All had PH with a Hepatic Venous pressure Gradient (HVPG) of 12 mmHg or higher [24].

Thirty-six patients were randomized to rifaximin-α 550 mg twice daily and eighteen to placebo with identical tablets in shape and size.

During LVC, 25 ml of blood was collected from the femoral artery at baseline and follow-up. Blood was immediately placed on ice; a fraction was centrifuged and both samples of full blood and EDTA plasma were stored at -80 degrees Celsius until analysis.

All patients gave written consent to participation. The trial was approved by the Danish Health Authorities (EudraCT 2012-002890-71), and registered in clinicaltrialsregister.eu on August 16, 2012. The study protocol conformed to the ethical guidelines of the 1975 Declaration of Helsinki and was approved by the Scientific Ethics Committee of the Capital Region of Denmark (H-1-2012-078) on August 20, 2012. The Good Clinical Practice unit (GCP unit) of Copenhagen University Hospital served as the external monitor of the trial, and initiated the trial on November 20, 2012.

The trial was further registered in clinicaltrials.gov (NCT1769040).

The first patient was enrolled and randomized on January 29, 2013. Last patient, last visit was performed on December 23, 2015 and the trial was closed as per protocol on December 30, 2015.

### sCD163 and sMR assays

Concentrations of sCD163 and sMR were determined by in-house sandwich enzyme-linked immunosorbent assays (ELISA)s using a BEP-2000 ELISA-analyzer (Dade Behring) essentially as previously described [25, 26]. Reference intervals of 0.69 to 3.86 mg/l for sCD163 and 0.10–0.43 mg/L for sMR in healthy individuals have previously been established with the same assays [26, 27].

Both substances are resistant to repeated freezing and thawing, and are stable for prolonged freezing at -80 degrees Celsius.

### Assays of PRO-C3, P4NP7S, C3M, and C4M

Neoepitope markers of collagen remodeling (type III and IV collagen, C3M and C4M) and collagen-formation (type III and IV, PRO-C3 and P4NPS7) were measured in plasma by well characterized assays as previously described [13, 14, 28, 29].

In brief, 96-well streptavidin plates were coated with biotinylated synthetic peptides specific for the fragments of interest and incubated for 30minutes at 20°C. 20ul of standard peptide or pre-diluted plasma were added to designated wells followed by addition of peroxidase-conjugated specific monoclonal antibodies and incubated for 1 hour at 20°C or 20 hours at 4°C (depending on the assay). Then, tetramethylbenzidine (TMB) was added to each well and plates were incubated for 15 minutes at 20°C. The enzymatic reaction was stopped by the addition of 0.18M H_2_SO_4_ and absorbance was measured at 450 nm with 650nm as a reference. All incubation steps included shaking of 300 rounds per minutes, and each incubation step was followed by five times washing with buffer (20mM Tris, 50mM NaCl, pH 7.2).

### Lipopolysaccharide binding protein (LBP)

LBP was measured in ethylene diamine tetra-acetic acid plasma with a commercially available ELISA (Vaiomer SAS, Toulouse, France). Protease activity was assessed by recording absorbance at 405 nm. The minimal detection limit was 3.5 μg/mL.

### Statistical analyses

Data was reported as median and range since data did not consequently follow a Gaussian normal distribution. Differences between rifaximin-α and placebo groups at baseline and follow-up were evaluated by Mann-Whitney test of delta values (the difference between follow up and baseline).P-values were calculated two-tailed, with four digits. Subgroup analyses were performed for participants with elevated LBP (n = 37). Spearman rank correlations on baseline values to identify associations between disease severity and biomarkers were performed. Data were handled using MedCalc Statistical Software (v 14.8.1 MedCalc Software bvba, Ostend, Belgium) and GraphPad (Software Prism 6.0.7, La Jolla, CA 92037 USA).

## Results

**Fig 1:**
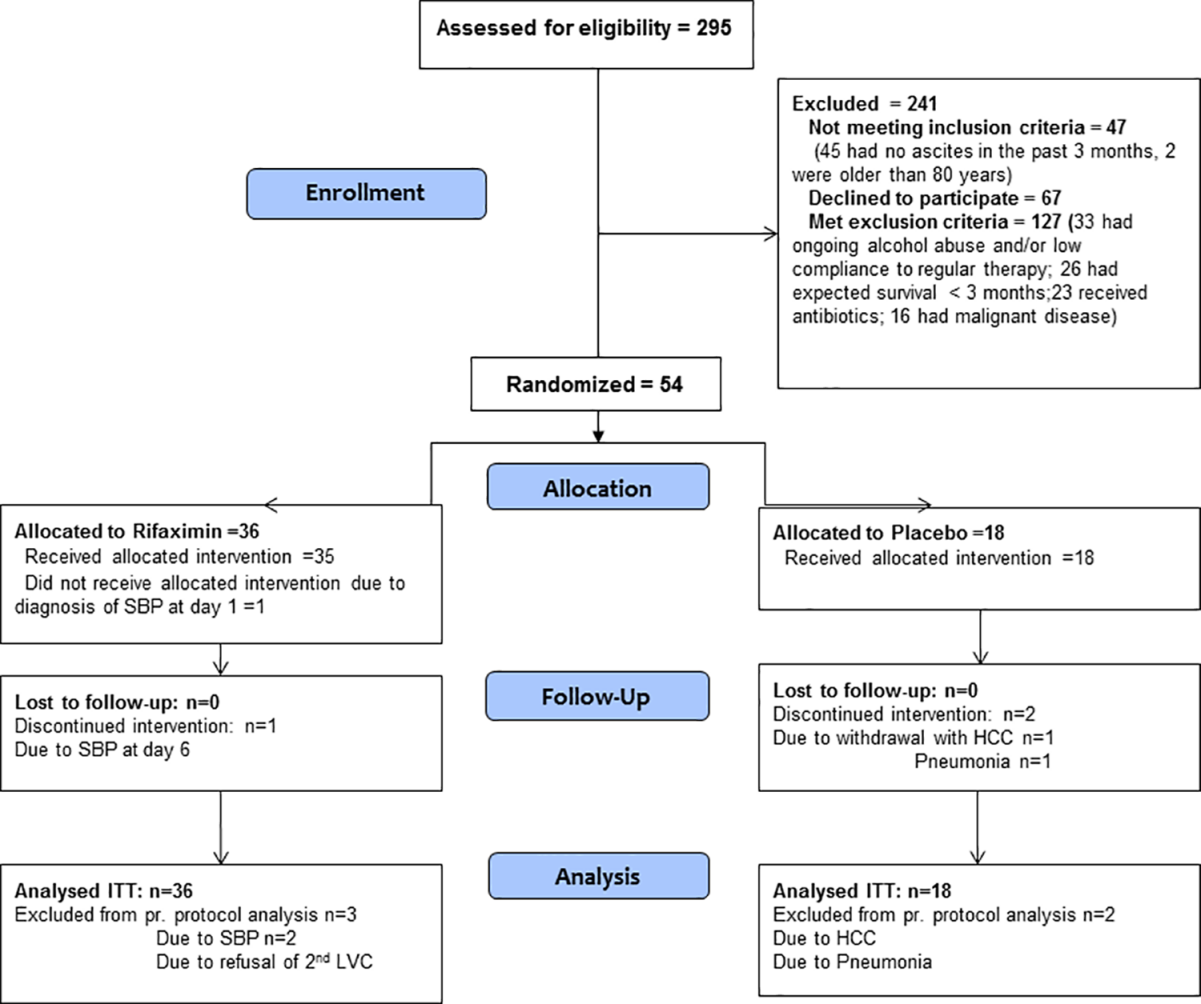
Trial flow diagram.

Baseline characteristics and demography in the two groups are shown in Table 1 and reported previously [23, 24]. Trial flow chart is reported previously and in Fig 1 [24].

**Table 1:** Demographic characteristics in the two groups (rifaximin-α and placebo) [24].

	Rifaximin-α	Placebo	p-value
**Sex** (male/female)	31/5	14/4	N/A
**Age**	58.5 (33–68)	52.5 (34–74)	0.2
**Child Class** B/C	27/9	17/1	N/A
**MELD**	12 (6–25)	9.5 (6–15)	0.007
Albumin g/l	28.5 (21–40)	32 (24–43)	0.16
Bilirubin μmol/l	24 (8–166)	16.5 (5–40)	0.002
Creatinine μmol/l	60 (43–171)	73 (44–146)	0.53
Platelets 10^9^/l	131 (27–562)	151.5 (56–275)	0.87
Hemoglobin mmol/l	7.6 (5.3–9.6)	7.85 (5–9.8)	0.36
Alanine transaminase U/l	25.5 (10–153)	28 (15–56)	0.79
Alkaline phosphatase U/l	121.5 (53–1200)	146 (47–459)	0.82

Values are given in median and minimum and maximum values, as they do not follow normal distribution, unless otherwise stated. MELD: Modified end stage liver disease score

**Fig 2:**
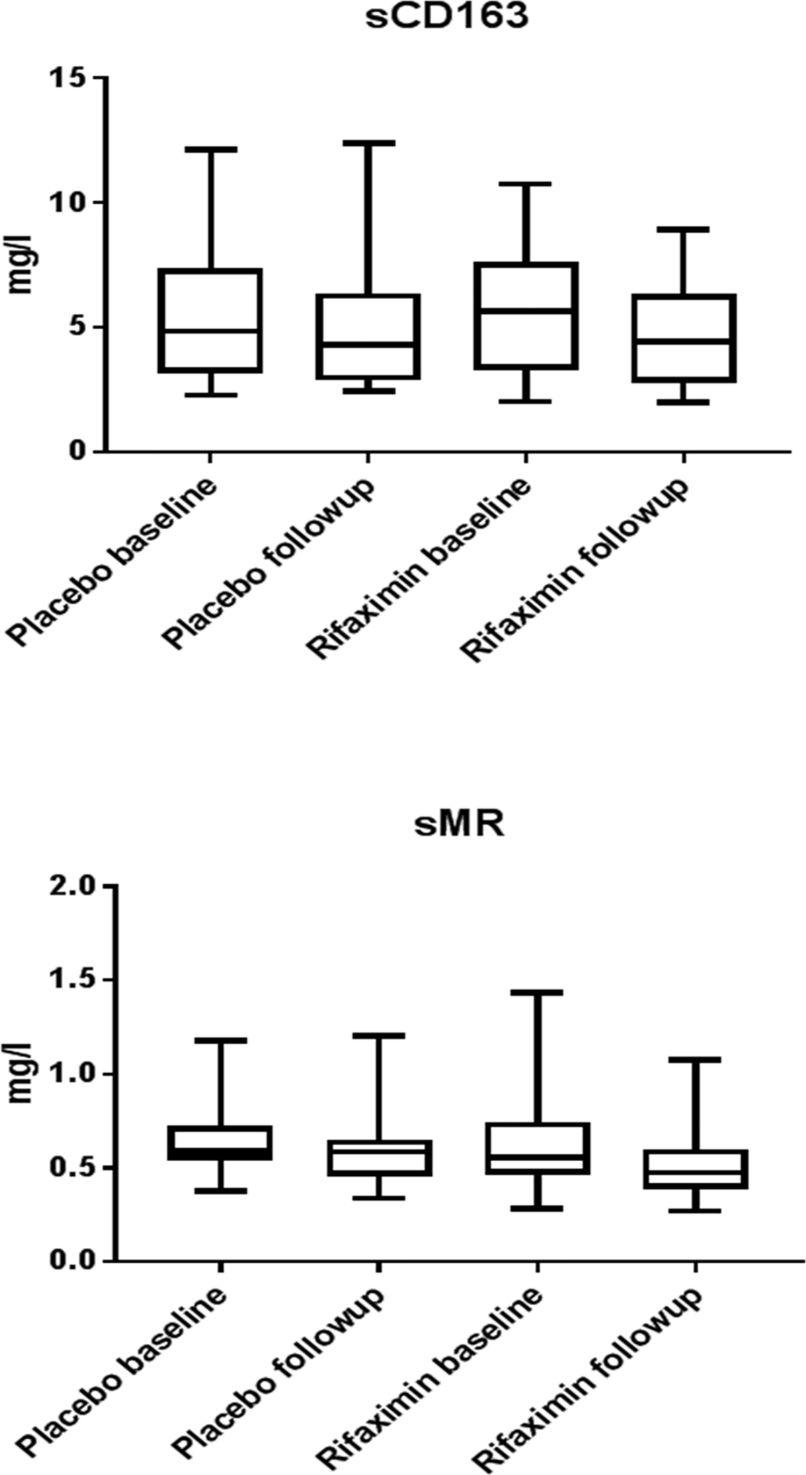
Levels of sCD163 and sMR in groups (rifaximin-α and placebo) before and after treatment.

Rifaximin-α had no effect on sCD163 (median (range) sCD163 5.64(2.02 to 10.8) at baseline versus 4.42(1.98 to 8.92) at follow-up in the rifaximin-α group and 4.85 (2.29 to 12.1) at baseline versus 4.32 (1.98 to 12.4) at follow-up in the placebo-group), p = 0.34, and 95% Confidence Interval of difference in median values -2.61 to 1.16; and no effect on sMR, (median (range) sMR 0.56 (0.28 to 1.43) at baseline versus 0.47 (0.37 to 1.08) at follow-up in the rifaximin-α group and 0.59 (0.38 to 1.18) at baseline versus 0.59 (0.34 to 1.2) at follow-up in the placebo group), p = 0.34, and 95% Confidence Interval of difference in median values -0.214 to 0.055; Fig 2.

**Fig 3:**
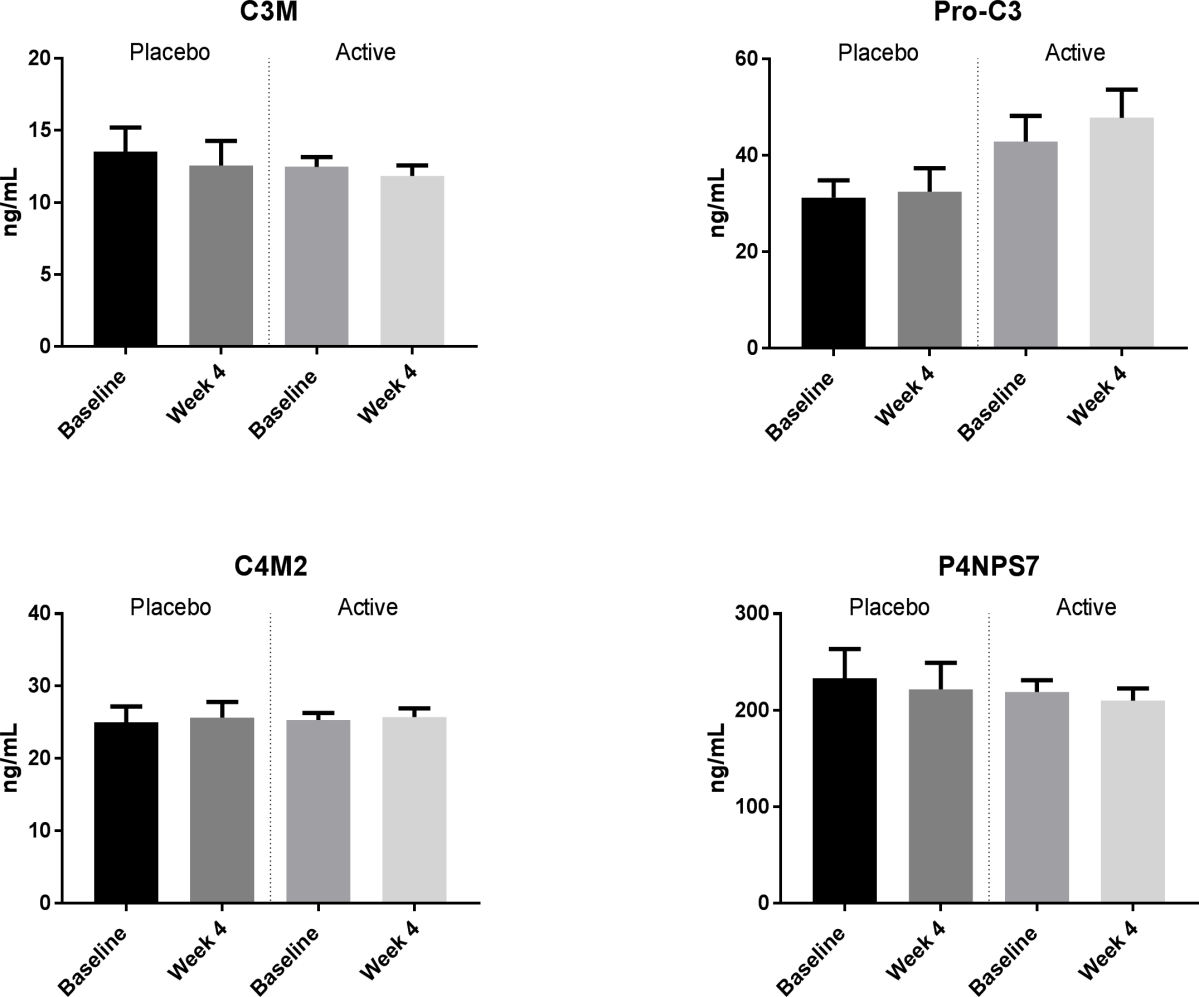
Levels of neoepitope markers of collagen remodeling at baseline and after 4 weeks of treatment.

No significant differences in markers of collagen formation and fibrogenesis were observed between the rifaximin-α group and placebo group at baseline or at follow-up, Fig 3, Table 2.

**Table 2:** Neoepitope markers of collagen remodeling before and after treatment.

	Placebo (n = 18)			p	Rifaximin-α (n = 18)			p
	Baseline	Follow-up	Delta		Baseline	Follow-up	Delta	
PRO-C3 (ng/ml)	26.8 (20.6 – 40.1)	30.6 (18.9 – 40.5)	-0.05 (-8.9–5.3)	0.95	34.5 (25.6 – 41.1)	35.9 (27.0 – 51.1)	3.9 (-0.7–9.1)	0.49
P4NPS7 (ng/ml)	182.3 (168.5 – 230.9)	186.7 (146.7 – 257.7)	-14.1 (34.5–20.4)	0.94	207.4 (186.5 – 246.1)	179.4 (164.5 – 253.5)	-7.2 (-28.2–11.0)	0.61
C3M (ng/ml)	11.5 (9.7 – 13.4)	10.17 (8.8–14.1)	-0.15 (-2.0–0.97)	0.63	12.5 (10.3 – 14.1)	11.4 (9.0–13.7)	-0.7 (-2.0–0.5)	0.53
C4M (ng/ml)	22.2 (19.38–28.9)	21.2 (19.1 – 32.3)	1.4 (-2.1–3.3)	0.76	24.1 (22.7 – 28.4)	25.1 (20.8 – 30.0)	-0.4 (-1.6–2.3)	0.87

Values of the collagen markers are given as median +/- percentile range 95%.

SCD163 was associated with liver disease severity (MELD score, rho = 0.42, p = 0.001 and Child score, rho = 0,27, p = 0.05). Both sCD163 and sMR was associated with low albumin levels (sCD163 rho = -0.47, p = <0.001 and sMR rho = -0,43, p = 0.001). However, sCD163 and sMR were not associated to portal hypertension or markers of bacterial translocation (for sCD163: HVPG rho = -0.075, p = 0.59; LBP rho = -0.078, p = 0.57 and LPS, rho = -0.042, p = 0.08. For sMR: HPVG, rho = 0.021, p = 0.88, LBP, rho = 0.008, p = 0.95, and LPS, rho = -0.202, p = 0.14). The neoepitope markers were not associated to either disease severity or markers of bacterial translocation, S1 Table.

We found no effect of rifaximin-α on neither sCD163 (p = 0.49), sMR (p = 0.052) nor the neoepitope markers in the subgroup of patients with elevated LBP (n = 37, 25 rifaximin group, 12 placebo group), S2 Table.

## Discussion

In this study the effects of rifaximin-α on macrophage activation markers sCD163 and sMR, and neoepitope markers of collagen remodeling were assessed in a randomized trial. We found no effect of rifaximin-α on any of the evaluated markers. However, increased levels of sCD163 were associated to disease severity and low albumin levels, but not to portal hypertension.

The macrophage activation marker sCD163 is associated with liver inflammation and fibrosis in chronic viral hepatitis B and C and is reduced after anti-viral treatment [8, 20, 30]. Further, sCD163 and sMR has been evaluated alone and in combination with fibrosis markers in predictive scores for clinically significant portal hypertension [9, 10], and also as a predictor of decompensation and mortality in cirrhosis [7, 31, 32]. Liver macrophages (Kupffer cells) express toll-like-receptors that respond to lipopolysaccharides (LPS) in blood. Presence of LPS and the LPS-binding protein LBP is supposed to be due to bacterial translocation and increased gut-blood permeability in patients with cirrhosis [33, 34]. In a previous study there was no association between sCD163 and LBP in patients selected for TIPS treatment. Interestingly, sCD163 levels were unchanged despite successful TIPS procedure and significant reductions in LBP levels [35]. A recently published study did contrariwise find an association between liver disease severity expressed as Child score and D’amico cirrhosis stage, and LBP and sCD14 [36]. In this cohort, levels of sCD163 and sMR were much higher in both patients with Child A, B and C, than demonstrated in the present study of 54 patients, and the prognostic value of sMR and sCD163 could not be confirmed. Further longitudinal studies in patients with progressing liver disease and with Child C are needed to explore this area. Basic studies have suggested that rifaximin-α may have a protective effect on bacterial translocation by increasing trans-epithelial resistance in the small intestine [37, 38].

If rifaximin-α prevents bacterial translocation, sCD163, as a marker of macrophage activation is expected to decrease during rifaximin-α treatment. However, our negative efforts to demonstrate such an effect is in line with our previous data from this randomized trial, where we found no effect of rifaximin-α on clinical parameters such as HVPG and cardiac output, no effect on biochemical parameters and inflammation markers (e.g. Tumor necrosis factor alpha and Interleukins 6, 10 and 18), and no effect on the composition of bacterial DNA in blood or stool [23, 24].

Previous studies have evaluated the significance of several neoepitope collagen markers as prognostic factors for portal hypertension in cirrhosis [16, 39]. Both C3M, C4M, PRO-C3 and P4NPS7 were reported to be associated with HVPG alone and in combination with the MELD score. These studies examined collagen markers in patients with varying severity of cirrhosis with MELD score in a range from 5 to 25. In the present study only P4NPS7 and C4M2 were significantly correlated to LPS, and none of the other markers of bacterial translocation or disease severity. We evaluated a homogenous group of patients with stable, but decompensated cirrhosis with a median MELD score of 12 (IQ range 7–16). Study participants were selected after presence of portal hypertension and the severely ill patients were excluded, which clearly narrowed the patient material to a highly selected group. This may explain the lack of association between neoepitope markers and disease severity in the present study. The pathophysiological mechanisms of rifaximin-α have been evaluated in several studies [40, 41]. Several factors may influence the lacking impact of rifaximin-α on collagen markers and fibrogenesis.

Patients were treated with rifaximin-α for 28 days and as previously reported we found no effect of rifaximin-α on systemic inflammation or bacterial composition in the gut, which indicates that the link between fibrogenesis and systemic inflammation is not affected by rifaximin-α. Also, in this patient cohort with stable but severe liver disease, the neo formation of fibrosis may have little influence on clinical and biochemical appearance of disease.

The beneficial effect of rifaximin-α in hepatic encephalopathy has been demonstrated in several studies, irrespective its unclear mechanism of action [21, 42]. In this randomized clinical trial of 54 patients with stable liver cirrhosis and ascites, we did not find a beneficial effect of rifaximin outside the current indication of overt hepatic encephalopathy. Future insight into the impact of bacterial translocation and leaky gut in cirrhosis on mechanisms of systemic inflammation is needed to evaluate indications for rifaximin-α outside hepatic encephalopathy and search for other treatment options in systemic inflammation. Also, further investigations into the possible effects of rifaximin on epithelial resistance and gut barrier functions as well as effects on fibrogenesis and fibrosis activity in liver disease is highly warranted.

This study was conducted as a double blind, placebo controlled randomized trial. The methodology may vouch for robust systematically collected data after applicable standards.

The study is of limited size though, and future trials may address selected areas of importance for systemic inflammation in the search for clinically relevant outcomes. Also assessment of drugs with different pharmacodynamics and targets may be applied in trials on systemic inflammation.

In conclusion, rifaximin-α treatment has no significant impact on macrophage activation in the liver as well as no effect on collagen markers. The effect of rifaximin-α on factors in the systemic inflammatory response theory is limited. Further studies to clarify both effects of rifaximin-α and the impact of the activated inflammation cascade in cirrhosis are in demand.

## Supporting information

S1 Checklist CONSORT 2010 checklist. (DOC)

S1 Table Correlation matrix between disease severity, bacterial translocation and neoepitope markers of collagen formation. (DOCX)

S2 Table Differences between groups in the subgroup of patients with elevated LBP. (DOCX)

S1 Approval Approval Danish Medicines Authority. (PDF)

S2 Approval Approval Scientific Ethics Committee. (PDF)

S1 Report Initiation report Good Clinical Practice Unit, Copenhagen University. (PDF)

S1 Protocol Protokol_version6.2. (DOC)

S1 Dataset Data set, fibrosis markers. (XLSX)

## Abbreviations

HVPG: Hepatic venous pressure gradient
LVC: Liver Vein Catheterization
LPS: Lipopolysaccharide
LBP: Lipopolysaccharide-binding protein
MELD: Modified Endstage Liver Disease model
PH: Portal Hypertension
sCD163: soluble CD163
sMR: soluble Mannose Receptor
