# Supplementary material for: No effect of rifaximin on soluble CD163, mannose receptor or type III and IV neoepitope collagen markers in decompensated cirrhosis: Results from a randomized, placebo controlled trial
Source: PLoS One. 2018 Sep 5;13(9):e0203200. doi: 10.1371/journal.pone.0203200 (PMC6124759; doi:10.1371/journal.pone.0203200)
Supplement: S1 Table — (DOCX) [file pone.0203200.s002.docx]

S1 table:

|  | C3M |  | Pro-C3 |  | P4NPS7 |  | C4M2 |  |
| --- | --- | --- | --- | --- | --- | --- | --- | --- |
|  | Rho | p | rho | p | rho | p | rho | p |
| MELD | 0.0789 | 0.5703 | 0.1382 | 0.3186 | 0.2146 | 0.1191 | 0.0667 | 0.6316 |
| Child | 0.0872 | 0.5304 | 0.0830 | 0.5504 | 0.1299 | 0.3488 | 0.1405 | 0.3106 |
| HVPG | -0.2332 | 0.0895 | -0.0725 | 0.6023 | -0.2207 | 0.1087 | -0.1086 | 0.4340 |
| Albu | -0.0953 | 0.4928 | -0.1201 | 0.3867 | -0.186 | 0.1759 | -0.209 | 0.1292 |
| sCD163 | 0.2119 | 0.1239 | 0.2533 | 0.0645 | 0.2595 | 0.0580 | 0.1143 | 0.4101 |
| sMR | 0.1674 | 0.2262 | 0.0772 | 0.5785 | 0.2533 | 0.0645 | 0.2293 | 0.0952 |
| LPS | -0.1950 | 0.1575 | -0.1113 | 0.4226 | -0.3063 | 0.0242 | -0.3111 | 0.0220 |
| LBP | -0.0285 | 0.8374 | -0.1390 | 0.3158 | -0.1675 | 0.2259 | -0.04313 | 0.7568 |

Spearman rank correlation matrix of neoepitope markers and markers of disease severity in cirrhosis and bacterial translocation.
