# Supplementary material for: No effect of rifaximin on soluble CD163, mannose receptor or type III and IV neoepitope collagen markers in decompensated cirrhosis: Results from a randomized, placebo controlled trial
Source: PLoS One. 2018 Sep 5;13(9):e0203200. doi: 10.1371/journal.pone.0203200 (PMC6124759; doi:10.1371/journal.pone.0203200)
Supplement: S2 Table — (DOCX) [file pone.0203200.s003.docx]

S2 table:

|  | Rifaximin-α^A^ baseline | Rifaximin-α follow-up | Placebo^B^ baseline | Placebo follow-up | p-value | Median CI^C^ |
| --- | --- | --- | --- | --- | --- | --- |
| sCD163 (mg/l) | 4.6 (2.0 – 11.0) | 4.6 (2.0 – 11.0) | 4.8 (2.4 – 12.0) | 4.5 (2.2 – 8.9) | 0.495 | -0.64 to 0.5 |
| sMR (mg/l) | 0.59 (0.52 - 0.86) | 0.53 (0.3 – 0.7) | 0.6 (0.28 – 1.4) | 0.5 (0.27 – 1.1) | 0.052 | -0.113 to 0.001 |

Data are given in median (min-max). P-values are analyzed as differences between baseline and follow-up.

**^A^** N=25

**^B^** N=12

**^C^** 95 % Confidence interval of difference in median values.
