# Supplementary material for: No effect of rifaximin on soluble CD163, mannose receptor or type III and IV neoepitope collagen markers in decompensated cirrhosis: Results from a randomized, placebo controlled trial
Source: PLoS One. 2018 Sep 5;13(9):e0203200. doi: 10.1371/journal.pone.0203200 (PMC6124759; doi:10.1371/journal.pone.0203200)
Supplement: S1 Approval — (PDF) [file pone.0203200.s004.pdf]

Sundhedsstyrelsen
Danish Health and Medicines Authority

Hvidovre Hospital, Gastroenterologisk afdeling
Att: Nina Kimer
Kettegård Allé 30
2650 Hvidovre

16. august 2012

T (dir.) +45 44 88 91 23
kf@dkma.dk

Journal no:
LMST2012073074

Vores ref: Marie-Louise Udengaard
Deres ref: Nina Kimer

"Effekt af Rifaximin på inflammation og hæmodynamik hos patienter med lever cirrose"
Protokol nummer: RifaxNK150612
EudraCT nummer: 2012-002890-71

Afgørelse:

Sundhedsstyrelsen giver tilladelse til, at det kliniske lægemiddelforsøg må udføres, jf. lægemiddellovens[1] § 88, stk. 1.

Tilladelsen gælder til og med **31-12-2014**

Forsøget omfatter følgende forsøgslægemidler:

- **Xifaxan (rifaximin), 550 mg tablet**
- **Placebo**

Det er et vilkår for denne tilladelse, at Sundhedsstyrelsen **orienteres**, hvis

- Forsøget forlænges udover datoen i tilladelsen
- Der indgår et nyt center eller dette ændres (opdateret xml-fil).
- Principal/koordinerende investigator ændres (opdateret xml-fil)
- CRO/ansøger ændres og
- Når forsøget afsluttes i Danmark.

På http://laegemiddelstyrelsen.dk/da/topics/bivirkninger-og-forsoeg/kliniske-forsoeg/forsoeg-med-mennesker/vejledning-til-ansoegning-om-tilladelse----mennesker/aendringer-til-kliniske-forsoeg fremgår det, hvilke ændringer, vi skal give tilladelse til.

[1] Lov nr. 1180 af 12. december 2005 om lægemidler, som ændret ved lov nr. 538 af 8. juni 2006 og lov nr. 1557 af 20. december 2006

Sundhedsstyrelsen
Axel Heides Gade 1
2300 København S
Denmark

T +45 72 22 74 00
E sst@sst.dk
www.sst.dk

Følgende er lagt til grund ved vores vurdering:

Dokumenter:

- Følgebrev dateret 02-07-2012/04-07-2012, underskrevet af Nina Kimer og Flemming Bendtsen
- Deltagerinformation / Samtykke- og fuldmagtserklæring, version 1.0, dateret 27-06-2012.
- Kontrakt mellem Norgine Danmark A/S og Sponsor
- Orienteringsbrev til fremstiller
- Aftale Sponsor og investigatorer imellem
- Aftale vedr. studiefaciliteterne
- Produktresumé for Xifaxan
- Produktresumé for Placebo
- Eksempler på etiketter
- Investigator's Brochure version 7.0, dateret juli 2011.
- Investigational medicinal product dossier (IMPD), dateret juni 2012.
- Brev med svar på begrundet indsigelse, dateret 14-08-2012
    - Ansøgningsskema. Pdf+ xml-fil.
    - Protokol RifaxNK150612**,** version 3, dateret 13-08-2012

Forsøget kan påbegyndes når der tillige foreligger en tilladelse fra en videnskabsetisk komité.

Der henvises til uddrag af lovgivning i vedlagte bilag.

Spørgsmål bedes rettet til Cand. Pharm Marie-Louise Udengaard på telefon: 4488 9187 eller e-mail: MLU@dkma.dk

Med venlig hilsen

Marie-Louise Udengaard
M. Sc. Pharm

Kopi: Til de videnskabsetisk komitéer for Region Hovedstaden

Lovmæssige forpligtigelser i forbindelse med kliniske lægemiddelforsøg.

God klinisk praksis (GCP)
Kliniske lægemiddelforsøg skal foregå i overensstemmelse med god klinisk praksis (GCP), jf. lægemiddellovens[2] § 88, stk. 2, og bekendtgørelse om god klinisk praksis i forbindelse med kliniske forsøg med lægemidler på mennesker[3].

God fremstillingspraksis (GMP)
Lægemidler til kliniske forsøg skal overholde gældende standarder for god fremstillingspraksis, jf. lægemiddellovens § 92, stk. 1, og bekendtgørelse om fremstilling og indførsel af lægemidler og mellemprodukter[4]. Forsøgslægemidler der fremstilles eller indføres fra et 3. land (ikke EU/EØS-land) skal overholde standarder for god fremstillingspraksis (mindst svarende til EU GMP).
For sikring af, at forsøgslægemidlerne fremstillet i 3. land overholder EU GMP eller tilsvarende, er det Sundhedsstyrelsens praksis, at der på forlangende skal kunne fremskaffes dokumentation herfor for fremstillersted(erne) i 3. land. Dette kan være i form af GMP-certifikat fra EU myndighed, og/eller EU GMP auditrapport fra en QP og/eller anden EU GMP myndighedsrapport. Dette gælder også for fremstillersted for aktive biologiske substanser. For lande med MRA aftale (Canada, Schweiz, Australien og New Zealand) kan ovenstående dokumentation erstattes af et GMP certifikat og/eller fremstillertilladelse udstedt af myndighed i det pågældende MRA land.

God distributionspraksis (GDP)
Sundhedsstyrelsen skal give tilladelse til bl.a. en gros- eller detailforhandling af lægemidler, dvs. distribution af lægemidler, jf. lægemiddellovens § 39, stk. 1, samt bekendtgørelse nr. 1243 af december 2005 om distribution af lægemidler.

Gratis udlevering af forsøgslægemidler
Forsøgslægemidler og eventuelle anordninger, der anvendes til at indgive forsøgslægemidler, skal udleveres gratis til forsøgspersoner, jf. Bekendtgørelse om god klinisk praksis i forbindelse med kliniske forsøg med lægemidler på mennesker § 13.

Ændringer i kliniske forsøg
§ 4 i bekendtgørelse om kliniske forsøg med lægemidler på mennesker anfører, hvornår der skal ansøges om Sundhedsstyrelsens tilladelse til at foretage ændringer i et forsøg. Se også ”Ændringer (amendments) til ansøgning om klinisk forsøg” på http://laegemiddelstyrelsen.dk/da/topics/bivirkninger-og-forsoeg/kliniske-forsoeg/forsoeg-med-mennesker/vejledning-til-ansoegning-om-tilladelse----mennesker/aendringer-til-kliniske-forsoeg.aspx

---

[2] Lov nr. 1180 af 12. december 2005 om lægemidler, som ændret ved lov nr. 538 af 8. juni 2006 og lov nr. 1557 af 20. december 2006
[3] Bekendtgørelse nr. 744 af 29. juni 2006 om god klinisk praksis i forbindelse med kliniske forsøg med lægemidler på mennesker
[4] Bekendtgørelse nr. 1242 af december 2005 om fremstilling og indførsel af lægemidler og mellemprodukter

Rapportering af bivirkninger i forsøget

Sponsor skal underrette Sundhedsstyrelsen

- *omgående* ved uventede og alvorlige formodede bivirkninger
- en gang årligt med en liste over alle alvorlige formodede indtrufne bivirkninger samt en rapport om forsøgspersonernes sikkerhed, jf. lægemiddellovens § 89, stk. 2.

Afslutning af forsøget

Sponsor skal underrette Sundhedsstyrelsen

- om forsøgets afslutning (senest 90 dage herefter)
- om forsøgets resultat (snarest muligt og inden 1 år efter afslutning)
- om afbrydelse af forsøget tidligere end planlagt (inden 15 dage). Der skal indsendes en begrundelse herfor, jf. lægemiddellovens § 89.
