# Supplementary material for: No effect of rifaximin on soluble CD163, mannose receptor or type III and IV neoepitope collagen markers in decompensated cirrhosis: Results from a randomized, placebo controlled trial
Source: PLoS One. 2018 Sep 5;13(9):e0203200. doi: 10.1371/journal.pone.0203200 (PMC6124759; doi:10.1371/journal.pone.0203200)
Supplement: S2 Approval — (PDF) [file pone.0203200.s005.pdf]

Region Hovedstaden

Koncern Organisation og Personale

De Videnskabsetiske Komiteer
i Region Hovedstaden

Kongens Vænge 2
3400 Hillerød

Telefon 38 66 63 95
Direkte 38 66 63 21
Mail vek@regionh.dk

Web www.regionhovedstaden.dk

EAN-nr: 5798001555203
Bank: Danske Bank 3100
3100142287
CVR/SE-nr: 29 19 06 23

Protokol nr.: H-1-2012-078

Ref.: Kristine Nordkvist

Dato: 20. august 2012

Læge
Nina Kimer
Hvidovre Hospital
Klinisk Fysiologisk og Nuklearmedicinsk afdeling afsnit 239
Kettegård Alle 30
2650 Hvidovre

Effekt af rifaximin på inflammation og hæmodynamik hos patienter med lever-cirrose.

Original titel: Intestinal decontamination with rifaximin. Effects on the inflammatory and circulatory state in patients with cirrhosis and ascites – A randomised controlled clinical study
Eudract nr.: 2012-002890-71

Den Videnskabsetiske Komite B for Region Hovedstaden har behandlet sagen på sit møde den 16. august 2012 og truffet følgende

Afgørelse:

Projektet godkendes i henhold til lov om et videnskabsetisk komitesystem**,** lov nr. 593 af 14. juni 2011 om videnskabsetisk behandling af sundhedsvidenskabelige forskningsprojekter.

Godkendelsen gælder for de anmeldte forsøgssteder, den anmeldte forsøgsansvarlige i Danmark samt for den angivne forsøgsperiode.

Godkendelsen gælder til den 31. marts 2015 og omfatter følgende dokumenter:

- Protokol, version 2, dateret 18. juli 2012,
- Retningslinjer for mundtlig deltagerinformation, version 1 og 2, dateret 6. juli 2012,
- Deltagerinformation, version 1, dateret 27. juni 2012,
- Samtykkeerklæring, fortryktversion (S4) fra Det Videnskabsetiske Komitésystem, påtrykt titel på forsøget,

Komiteen har endvidere set følgende dokumenter:
- Fuldmagt, version 1, dateret 27. juni 2012,
- Investigational programme, dateret 27. juni 2012,
- Clinical trial agreement,
- Bilag vedrørende økonomi og publikation, dansk udgave, oversættelse af afsnit fra ovenstående.,
- Protokolresumé, dateret 6. juli 2012.

Iværksættelse af projektet i strid med godkendelsen kan straffes med bøde eller fængsel, jf. komitélovens § 41.

Det er en betingelse for projektets iværksættelse, at Sundhedsstyrelsen også godkender forsøget. Vær opmærksom på, at godkendelsesperioden i Sundhedsstyrelsens afgørelse kan være en anden end i denne afgørelse. Det er forsøgsansvarliges ansvar at sikre – evt. ved ansøgning om forlængelse - at der hele tiden under projektets forløb er fornøden godkendelse fra de to myndigheder.

Ændringer*:*

Foretages der væsentlige ændringer i protokolmaterialet under gennemførelsen af projektet, skal disse anmeldes til komiteen i form af tillægsprotokoller. Ændringerne må først iværksættes efter godkendelse fra komiteen, jf. komitélovens § 27, stk. 1.

Anmeldelse af tillægsprotokoller skal ske elektronisk på www.drvk.dk med det allerede tildelte anmeldelsesnummer og adgangskode.

Væsentlige ændringer er bl.a. ændringer, der kan få betydning for forsøgspersonernes sikkerhed, fortolkning af den videnskabelige dokumentation, som projektet bygger på samt gennemførelsen eller ledelsen af projektet. Det kan fx være ændringer i in- og eksklusionskriterier, forsøgsdesign, antal forsøgspersoner, forsøgsprocedurer, behandlingsvarighed, effektparametre, ændringer om de forsøgsansvarlige eller forsøgssteder samt indholdsmæssige ændringer i det skriftlige informationsmateriale til forsøgspersonerne.

Hvor nye oplysninger betyder, at forskeren overvejer at ændre proceduren eller stoppe forsøget, skal komiteen orienteres om det.

Bivirkninger:

Løbende indberetning

Komiteen skal omgående underrettes, hvis der under projektet optræder formodet alvorlige, uventede bivirkninger (SUSARS) jf. komitélovens § 30, stk. 1 Indberetningen skal ledsages af kommentarer om eventuelle konsekvenser for forsøget. Pligten til indberetning omfatter SUSARS, der er forekommet i Danmark.

Indberetning af SUSARS til komitesystemet skal indholdsmæssigt følge vejledning om ansøgning om tilladelse til kliniske forsøg, afsnit 12.1 og 12.3 om indberetning af bivirkninger.

Årlig indberetning

Én gang årligt i hele forsøgsperioden skal komiteen have tilsendt en liste over alle formodet alvorlige (ventede og uventede) bivirkninger, som er indtruffet i forsøgsperioden (ASR/DSUR) sammen med en rapport om forsøgspersonernes sikkerhed, jf. komitélovens § 30, stk. 2.

Den årlige Indberetning skal i øvrigt følge vejledning om ansøgning om tilladelse til kliniske forsøg, afsnit 12.4 om indberetning af bivirkning.

Indberetningens form

Komiteen skal have tilsendt SUSARS og årlige indberetninger i form af rapport i pdf-format. Indberetningen skal ske elektronisk (krypteret) eller ved indsendelse af CD-rom.

Ved indberetning kan anvendes et skema, som kan findes på www.dnvk.dk. Skemaet indsendes elektronisk ved anvendelse af digital signatur.

Afslutning:

Den forsøgsansvarlige skal senest 90 dage efter afslutningen af projektet underrette komiteen herom, jf. komitélovens § 31, stk. 1. Projektet regnes som afsluttet, når sidste forsøgsperson er afsluttet.

Afbrydes projektet tidligere end planlagt, skal en begrundelse herfor sendes til komiteen senest 15 dage efter, at beslutningen er truffet, jf. komitélovens § 31, stk. 2.

Hvis projektet ikke påbegyndes, skal dette samt årsagen hertil meddeles komiteen.

Komiteen beder om kopi af den afsluttende forskningsrapport eller publikation, jf. komitelovens § 28, stk. 2.. Vi skal i den forbindelse gøre opmærksom på, at der er pligt til at offentliggøre både negative, positive og inkonklusive forsøgsresultater, jf. komitélovens § 20, stk. 1, nr. 8.

Tilsyn:
Det er Sundhedsstyrelsen, der fører tilsyn med lægemiddelforsøg.

Følgende komitemedlemmer deltog i mødebehandlingen:
Mikael Bitsch, Hanne Andersen, Else Marie Klærke, Jens Mørk Lauridsen, Henri Goldstein, Henrik Larsson og Lisbeth Tranebjærg.

Med venlig hilsen

Mikael Bitsch
Formand for Komite B

Kopi til:

- Sundhedsstyrelsen, Axel Heides Gade 1, 2300 København S
