# Supplementary material for: No effect of rifaximin on soluble CD163, mannose receptor or type III and IV neoepitope collagen markers in decompensated cirrhosis: Results from a randomized, placebo controlled trial
Source: PLoS One. 2018 Sep 5;13(9):e0203200. doi: 10.1371/journal.pone.0203200 (PMC6124759; doi:10.1371/journal.pone.0203200)
Supplement: S1 Report — (PDF) [file pone.0203200.s006.pdf]

GCP-enhedens godkendelse til forsøgsstart

**Protokol titel + versionsnr.**	Intestinal decontamination with rifaximin.<br>Effects on the inflammatory and circulatory state in patients with cirrhosis and ascites - A randomised controlled clinical study. Version 4		
**Sponsor**	Professor, overlæge, Dr. Med Flemming Bendtsen		
**Center**	Gastroenterologisk afdeling, Hvidovre	**EudraCT nr.**	2012-002890-71
**Investigator**	Læge Nina Kimer	**Internt projektnr.**	2012-483

Kommentar:
Godekendelse givet under forudsætning af at der følges hurtigt op på anmeldelsen af databehandling, samt at NK kontakter JB når hun har modtaget medicinen, så faktorer som antal, holdbarhed og etikettering, kodekuverter kan afklares.

GCP-enheden har, som et led i monitorering af forsøget, gennemført et initieringsbesøg den 19.11.2012, beskrevet i rapport nummer 1.

Det er konstateret, at forudsætningerne for at begynde forsøget på centret er til stede, se dog kommentarer.

GCP-koordinator: Jeanette Blom
(Jeanette Blom)

20/11 - 2012
(dato/underskrift)

Original til: Flemming Bendtsen
Kopi til: investigator NK,og GCP-enheden.
Godkendelsen arkiveres i forsøgets Trial Master File

Københavns Universitetshospitals
GCP | ENHED

Professor, overlæge, Dr. Med Flemming Bendtsen
Gastroenterologisk afdeling, afsnit 360,
Hvidovre Hospital
Kettegaard Alle 30
2650 Hvidovre

Bispebjerg Bakke 23, Bygning 51, 3.sal
2400 København NV
Tlf.: 35313893
Fax.: 35 31 38 89
E-mail.: jeanette.blom@regionh.dk
www.gcp-enhed.dk

20. november 2012

Vedr.: Intestinal decontamination with rifaximin

Kære Flemming

Hermed initieringsrapporten fra mit besøg i går mandag, samt tilladelse til opstart. Rapporten skal underskrives og gemmes i Trial Master File. Jeg vedlægger også vores spørgeskema vedrørende tilfredshedsundersøgelse. Måske er det Nina der skal udfylde det, da det helst skal udfyldes af den vi har haft mest kontakt med. På forhånd tak!

Ved spørgsmål eller lignende er du meget velkommen til at kontakte mig.

Venlig hilsen

Jeanette Blom

Jeanette Blom
GCP-koordinator

Initieringsrapport for initiering hos sponsor-investigator

**Protokol**	Intestinal decontamination with rifaximin.<br>Effects on the inflammatory and circulatory state in patients with cirrhosis and ascites - A randomised controlled clinical study		**Internt projektnr.**	2012-483
**Center**	Gastroenterologisk afdeling, Hvidovre		**EudraCT nr.**	2012-002890-71
**Sponsor**<br>**Investigator**	Professor, overlæge, Dr. Med Flemming Bendtsen<br>Læge Nina Kimer		**Rapport nr.**	1
**Deltagere**	***Navn (initialer)***	***Funktion***	**Dato for besøg**	19/11-12
	**Fra centret:**<br>Læge Nina Kimer	Investigator	**Dato for rapport**	19/11-12
			**GCP-koordinator**	J.B
	**Fra GCP-enhed:**<br>Jeanette Blom	GCP-koordinator	Intern review i GCP-enheden:<br>Initialer:	Dato:

Afkrydsning i felter med gråskravering kræver en kommentar.

**1. Protokol og amendments**	Ja	Nej	Ikke relevant	Ikke checket
1.1 Er protokollen/amendments dateret og signeret af sponsor-investigator?	☐	☒	☐	☐
1.2 Foreligger der accept til at udføre forsøget fra afdelings-/forskningsledelse?	☒	☐	☐	☐
1.3 Er der vejledt om at kontrakt mellem sponsor og lægemiddelvirksomhed bør vurderes juridisk?	☒	☐	☐	☐
1.4 Foreligger skriftlig aftale om uddelegering af sponsoropgaver?	☐	☐	☒	☐

Kommentarer:

1.1) Skal gøres

1.3) Der er underskrevet kontrakt med Norgine vdr, levering af lægemidlet. Den har været vurderet af VIF.

**2. Tilladelser**	Ja	Nej	Ikke relevant	Ikke checket
2.1 Foreligger anmeldelserne og er der givet tilladelse til forsøget fra:				
• Lægemiddelstyrelsen incl. EudraCT	☒	☐	☐	☐
• Den Videnskabsetiske Komité	☒	☐	☐	☐

2. Tilladelser	Ja	Nej	Ikke relevant	Ikke checket
• Datatilsynet	☐	☒	☐	☐
• Arbejdstilsynet	☐	☐	☒	☐
• Andre myndigheder	☐	☐	☒	☐
Notér detaljer under "kommentarer"				
2.2 Ved multicenterforsøg: Er centret omfattet af tilladelserne?	☐	☐	☒	☐
2.3 Er Multipraksisudvalget informeret (forsøg med praktiserende læger)?	☐	☐	☒	☐
2.4 Er der vejledt om regler for tilladelse til at have tilknytning til lægemiddelvirksomhed (hvis relevant)?	☐	☐	☒	☐

Kommentarer:

2.1

Lægemiddelstyrelsens godkendelse, dateret: 16.08.2012

Godkendelse baseret på følgende dokumenter:

Dokument	Version
Forsøgsprotokol	3 (står ikke i tilladelsen, men af følgebrev fremgår at version 3 er indsendt) + 4 indsendt 31.08.2012 med ændringer
Deltagerinformation	1(27.06.2012)
Samtykke/fuldmagtserklæringer	1(27.06.2012)
Andre, specificer	Div. dokumenter

Dato for afslutning af forsøget ifølge tilladelse: 31.12.2014

Videnskabsetisk Komités godkendelse, dateret: 20.08.2012 Journalnr: H-1-2012-078

Godkendelse baseret på følgende dokumenter:

Dokument	Version
Forsøgsprotokol	2, samt 4 (eftersendt og godkendt 10.09.2012)
Lægmandsbeskrivelse	06.07.2012
Deltagerinformation	1(27.06.2012) + 2 (22.08.2012)
Samtykke/fuldmagt	1(27.06.2012)
Andre, specificer	Div. dokumenter

Dato for afslutning af forsøget ifølge tilladelse: 31.03.2015

Datatilsynet:

Offentlig anmeldelse, dateret: anmeldelse afsendt: 12.11.2012. Behandling af anmeldelse pågår, da der har rejst sig nogle spørgsmål til databasen. Der er udformet databehandler aftale med SSI. N.K skal høre om der skal laves databehandleraftale med firma som leverer e-crf. Glostrup og RH samt GCP-enheden bør angives som databehandlere.

3. Projektdeltagere	Ja	Nej	Ikke relevant	Ikke checket
3.1. Er der udarbejdet en liste over uddelegering af forsøgsrelaterede opgaver med signaturer for relevante projektdeltagere?	☒	☐	☐	☐
3.2. Forefindes relevant dokumentation for projektdeltageres kvalifikationer (fx CV'er)?	☒	☐	☐	☐
3.3. Sikres det, at alle projektdeltagere er informeret om forsøget?	☒	☐	☐	☐

Kommentarer:

3.1) Ifølge listen må alle læger underskrive CRF. Det skyldes at e-crf er udformet således at den ikke som sådan underskrives samlet, men at hver læge underskriver som den der udfylder de pågældende data.

A.K har ikke fået uddelegeret opgaver, men har underskrevet. Det skyldes at han holder op i afdelingen pr. 1/1-2013. N.K er orienteret om at han skal påføres listen såfremt han skal have opgaver i forsøget inden.

3.2) C.V på F.B og S.M er ikke underskrevet og indeholder ikke oplysninger om GCP erfaring. Det oplyses af N.K at begge har praktisk erfaring med at køre GCP studier. Kan med fordel påføres CV.

4. Ressourcer	Ja	Nej	Ikke relevant	Ikke checket
4.1. Er de fornødne ressourcer til at gennemføre forsøget tilstede?	☒	☐	☐	☐
4.2. Er der udarbejdet et budget- og finansieringsforslag?	☒	☐	☐	☐
4.3. Er der indgået forsikringsaftaler (fx internationale forsøg med dansk sponsor)?	☐	☐	☒	☐

Kommentarer:

5. Aftaler	Ja	Nej	Ikke relevant	Ikke checket
5.1. Er der indgået aftale med relevante samarbejdspartnere?				
SSI	☐	☒	☐	☐
Apotek	☒	☐	☐	☐
Medicinalfirma, Norgine	☒	☐	☐	☐
Andre: Klin. Fysiologisk og Nuklearmedicinsk afd	☒	☐	☐	☐
Klinisk Forskningscenter, Hvidovre	☒	☐	☐	☐

Kommentarer:

5.1) Der er ikke endnu indgået en egentlig samarbejdsaftale med dem der leverer CRF, aftalen er indgået via mail.

Der skal indgåes aftale med SSI før prøverne sendes af sted til analyse. Aftale med Klinisk Forskningscenter skal underskrives.

6. Faciliteter/apparatur	Ja	Nej	Ikke relevant	Ikke checket
6.1 Er der hensigtsmæssige faciliteter?	☒	☐	☐	☐
6.2 Er nødvendigt apparatur tilgængeligt?	☒	☐	☐	☐

6. Faciliteter/apparatur	Ja	Nej	Ikke relevant	Ikke checket
6.3 Er der udarbejdet forskrifter for anvendelse af relevant apparatur?	☒	☐	☐	☐
6.4 Er der oprettet kvalitetssikringsprocedurer, herunder kalibreringsprocedurer, for relevant apparatur?	☒	☐	☐	☐
6.5 Er nødvendige tiltag for at sikre sammenlignelige resultater etableret (relevant ved multicenterforsøg, og hvor der er mere end et apparat til undersøgelse af det samme)?	☐	☐	☒	☐

Kommentarer:

6.3 + 6.4) Alle undersøgelser er standard og foretages efter hospitalets retningslinjer/instrukser. Der er udfærdiget SOP for procedure for blodprøvetagning undervejs under katerisation.

7. Laboratorieprøver	Ja	Nej	Ikke relevant	Ikke checket
7.1 Er laboratoriet, hvor lokalt udførte analyser bestemmes, besøgt af GCP-koordinator (fx ved ikke rutineprøver eller ved primær effektparameter)?	☐	☐	☒	☐
7.2 Er laboratoriet, hvor centralt udførte analyser bestemmes, besøgt af GCP-koordinator (fx ved ikke rutineprøver eller ved primær effektparameter)?	☐	☐	☒	☐
7.3 Er der procedurer for håndtering (fx nedfrysning, forsendelse etc.) af relevante laboratorieprøver?	☒	☐	☐	☐
7.4 Føres der optegnelser over opbevarede prøver?	☒	☐	☐	☐
7.5 Findes referenceværdier for relevante analyser for alle centre?	☒	☐	☐	☐
7.6 Findes metodebeskrivelse og dokumentation for kvalitetskontrol for ikke rutineanalyser?	☒	☐	☐	☐
7.7 Er nødvendige tiltag for at sikre sammenlignelige resultater etableret (relevant ved multicenterforsøg, og hvor der er mere end et apparat til analyse af det samme)?	☐	☐	☒	☐

Kommentarer:

7.1-7.4) Klin. Fysiologisk og Nuklearmedicinsk afd. På Hvidovre står for prøvetagning og håndtering, er vant til at køre kliniske forsøg og har faste procedurer for håndtering af prøverne. Det er ikke nødvendigt at allokere specielle folk derfra til forsøget.

7.5) De relevante referenceværdier er indsat i TMF.

7.6) Findes til dels i TMF og dels på de enkelte laboratorier. Der anvendes kun hospitalslaboratorier og SSI.

8. Forsøgspersoner	Ja	Nej	Ikke relevant	Ikke checket
8.1 Registreres forsøgspersonerne i sygehusets dokumentationssystem?	☒	☐	☐	☐
8.2 Er forsøgspersoner patienter med en patientjournal?	☒	☐	☐	☐
8.3 Er krav til journalføring ved deltagelse i klinisk forsøg drøftet?	☒	☐	☐	☐
8.4 Er rekrutteringsprocedure beskrevet?	☒	☐	☐	☐
8.5 Er der beskrevet retningslinjer for mundtlig deltagerinformation?	☒	☐	☐	☐
8.6 Findes et eksemplar af alt skriftligt materiale, som udleveres til forsøgspersonen fx:				

8. Forsøgspersoner	Ja	Nej	Ikke relevant	Ikke checket
Deltagerinformation inkl. samtykke-/fuldmagtserklæring	☒	☐	☐	☐
Tillægget "Forsøgspersoners rettigheder i et biomedicinsk forskningsprojekt" eller "Før du beslutter dig"	☒	☐	☐	☐
Deltagerkort	☐	☐	☒	☐
Annoncetekst	☐	☐	☒	☐
Standardbreve	☐	☐	☒	☐
Dagbog	☐	☐	☒	☐
Spørgeskemaer	☐	☐	☒	☐
Andet: medicindagbog	☒	☐	☐	☐
8.7 Vil forsøgspersonens egen læge som udgangspunkt blive orienteret?	☒	☐	☐	☐
8.8 Er der udarbejdet en screeningslog for forsøgspersoner?	☒	☐	☐	☐
8.9 Er der udarbejdet en patientidentifikationsliste?	☒	☐	☐	☐
8.10 Er der udarbejdet en kronologisk liste?	☐	☐	☒	☐

Kommentarer:

8.4) Der er indført ændring for nylig, således at N.K tager ud på andre hospitaler i regionen og informerer potentielle forsøgskandidater.

8.6) Der hører forskningsbiobank til fremtidig forskning til projektet. Patienten kan dog også frabede sig at deltage i dette. Vi drøfter om det kunne være en fordel at dokumentere patientens ja/nej til at afgive prøver til fremtidig forskning.

8.7) Via epikrise

9. Forsøgsmedicin	Ja	Nej	Ikke relevant	Ikke checket
9.1 Er der procedurer for modtagelse/håndtering/opbevaring/distribution/ destruktion af forsøgsmedicin?	☒	☐	☐	☐
9.2 Er der procedurer for samlet medicinregnskab på centret?	☒	☐	☐	☐
9.3 Er der procedurer for medicinregnskab/compliance for hver forsøgsperson?	☒	☐	☐	☐
9.4 Er der hensigtsmæssige opbevaringsfaciliteter for forsøgsmedicin på centret?	☒	☐	☐	☐
9.5 Føres temperatur logs?	☐	☒	☐	☐
9.6 Findes IB/produktresumé for forsøgsmedicin?	☒	☐	☐	☐
9.7 Er forsøgsmedicin etiketteret korrekt til klinisk forsøg?	☐	☐	☐	☒
9.8 Findes kopi af etiketter?	☒	☐	☐	☐
9.9 Findes forsendelseskvitteringer for modtaget forsøgsmedicin?	☐	☐	☐	☒
9.10 Er randomiseringsprocedure beskrevet?	☒	☐	☐	☐
9.11 Findes randomiseringsliste (sponsor eller tredjepart)?	☒	☐	☐	☐
9.12 Opbevares kodekuverter hensigtsmæssigt?	☐	☐	☐	☒
9.13 Er der beskrevet en procedure for kodebrud?	☒	☐	☐	☐

9. Forsøgsmedicin	Ja	Nej	Ikke relevant	Ikke checket
9.14 Er apoteket besøgt af GCP-koordinator?	☐	☐	☒	☐
9.15 Hvis relevant, foreligger der dokumentation for, at fremstiller er godkendt til at producere lægemidler (Ikke kendte fremstillere, §39-tilladelse)? (Ikke relevant hvis medicinen leveres til afdelingen af et sygehusapotek)	☐	☐	☒	☐
9.16 Foreligger relevant dokumentation for produktion af forsøgsmedicin i henhold til GMP? (Ikke relevant hvis medicinen leveres til afdelingen af et sygehusapotek)	☐	☐	☒	☐
9.17 Foreligger relevante analyse-/frigivelsescertifikater? (Ikke relevant hvis medicinen leveres til afdelingen af et sygehusapotek)	☐	☐	☒	☐

Kommentarer:

9.1) Vi har gennemgået proceduren i dag. Findes ikke skriftlig.

9.5) Kommer til at stå i rum hvor temperatur ikke overstiger 25 grader

9.7+9.9+9.12) Medicin leveres først senere i dag fra apoteket.

9.11) Findes på regionens apotek, Herlev

10. CRF	Ja	Nej	Ikke relevant	Ikke checket
10.1 Er der udarbejdet en CRF?	☒	☐	☐	☐
10.2 Foreligger der en entydig procedure for, hvorledes data føres ind i CRF og hvorledes rettelser foretages, dateres og signeres?	☐	☒	☐	☐
10.3 Findes en kopi af CRF'en i Trial Master File?	☐	☒	☐	☐
10.4 Foreligger der en procedure for regnskab med CRF'er (hvis relevant)?	☐	☐	☒	☐

Kommentarer:

10.2) Elektronisk CRF

10.3) Der foreligger i stedet komplet liste over indhold på hver side i CRF, version 4.

11. Kildedokumentation	Ja	Nej	Ikke relevant	Ikke checket
11.1. Er der udarbejdet et dokument, som specificerer kildedata (kildedataliste)?	☒	☐	☐	☐

Kommentarer:

12. Håndtering af alvorlige bivirkninger og hændelser	Ja	Nej	Ikke relevant	Ikke checket
12.1 Findes skriftlige procedurer for håndtering af SAE'er:				
- fra investigator til sponsor	☒	☐	☐	☐
- fra sponsor til investigator	☐	☐	☒	☐
- til Lægemiddelstyrelsen	☒	☐	☐	☐
- til andre landes lægemiddelstyrelser	☐	☐	☒	☐
- til evt. medicinalfirma	☒	☐	☐	☐

12. Håndtering af alvorlige bivirkninger og hændelser	Ja	Nej	Ikke relevant	Ikke checket
12.2 Findes relevante anmeldelsesblanketter?	[X]	[ ]	[ ]	[ ]
12.3 Er der beskrevet en procedure for udarbejdelse af årlige lister/rapport over ventede og uventede alvorlige bivirkninger til Lægemiddelstyrelsen og evt. andre relevante myndigheder, samt alvorlige bivirkninger og hændelser til VEK?	[X]	[ ]	[ ]	[ ]

Kommentarer

12.3) I protokol

13. Trial Master File	Ja	Nej	Ikke relevant	Ikke checket
13.1 Er der etableret en Trial Master File?	[X]	[ ]	[ ]	[ ]
13.2 Findes rapporter over gennemgang af projektbeskrivelsen?	[ ]	[X]	[ ]	[ ]
13.3 Findes der en besøgslog for monitor?	[ ]	[X]	[ ]	[ ]
13.4 Findes et eksemplar af ICH-GCP guideline?	[ ]	[ ]	[X]	[ ]

Kommentarer:

13.2) Vejledning foregået over mail. Der er ikke udfærdiget egentlig rapport.

13.3) Skal indsættes. Underskrives næste gang.

13.4) Tilgængelig online.

14. Datahåndtering	Ja	Nej	Ikke relevant	Ikke checket
14.1. Sikres hensigtsmæssig fysisk/elektronisk opbevaring af data, herunder CRF?	[X]	[ ]	[ ]	[ ]

Kommentarer:

14.1) TMF står på aflåst kontor.

15. Monitoreringsprocedure	Ja	Nej	Ikke relevant	Ikke checket
15.1 Er der indgået aftale mellem GCP-enheden og sponsor?	[X]	[ ]	[ ]	[ ]
15.2 Er den fremtidige monitoreringsplan beskrevet?	[X]	[ ]	[ ]	[ ]
15.3 Er der fulgt op på bemærkninger fra projektgennemgang, hvis relevant?	[X]	[ ]	[ ]	[ ]
15.4 Er rekrutteringshastighed drøftet?	[X]	[ ]	[ ]	[ ]
15.5 Er første monitoreringsbesøg planlagt?	[ ]	[X]	[ ]	[ ]
15.6 Er der udarbejdet et system, der sikrer at relevante data er monitoreret før de sendes til indtastning?	[X]	[ ]	[ ]	[ ]

Kommentarer:

15.2) Detaljeret monitoreringsplan bliver udfærdiget efter 1. monitoreringsbesøg.

15.5) J.B kontaktes efter 1. patient er indgået.

15.6) Tastes direkte ind i e-crf og monitoreres her. N.K undersøger monitoreringsadgang.

16. Registrering med henblik på publikation	Ja	Nej	Ikke relevant	Ikke checket
16.1. Er forsøget registreret i en offentligt tilgængelig database iht. retningslinierne fra ICMJE?	☒	☐	☐	☐

Kommentarer:

16.1) Er anmeldt via eudraCT

17. Andet, specificer
Kommentarer:

18. Bilag	Ja	Nej
18.1. Findes der bilag til denne rapport *(hvis ja, specificer under kommentarer)?*	☐	☒

Kommentarer:

19. Punkter til opfølgning

Dato	Opfølgning	Ansvar	Udført
19/11-12	Protokol skal underskrives (fx på forsiden)	NK/FB	
19/11-12	Høre om der skal laves databehandleraftale med firma som leverer e-crf., samt påføre Glostrup, RH og GCP-enheden som databehandlere.	NK	
19/11-12	FB og SM evt opdatere CV med GCP erfaring. Underskrive CV	FB/SM	
19/11-12	Aftale med Klinisk Forskningscenter skal underskrives.	NK	
19/11-12	Monitorlog indsættes i TMF	NK	
19/11-12	Undersøge monitoradgang til e-crf	NK	

20. Status	Ja	Nej
20.1. Er godkendelse til forsøgsstart udstedt?	☒	☐

Kommentarer:

20.1) Under betingelse af at der følges op på ovenstående og NK mailer til JB ang. modtaget forsøgsmedicins udseende, antal og holdbarhed.

Underskrifter

Rapporten er udfærdiget af:

Jeanett Blom

GCP-koordinator

20/11-2012

Dato

**Underskrifter**	
Som sponsor- investigator har jeg gennemgået rapporten og tager ansvar for evt. opfølgning	
[signature]	22/11-2012
Sponsor- investigator	Dato
Kopi til: Investigator NK, GCP-enheden.	
Den underskrevne rapport arkiveres i forsøgets Trial Master File	
