# Supplementary material for: No effect of rifaximin on soluble CD163, mannose receptor or type III and IV neoepitope collagen markers in decompensated cirrhosis: Results from a randomized, placebo controlled trial
Source: PLoS One. 2018 Sep 5;13(9):e0203200. doi: 10.1371/journal.pone.0203200 (PMC6124759; doi:10.1371/journal.pone.0203200)
Supplement: S1 Protocol — (DOC) [file pone.0203200.s007.doc]

Intestinal decontamination with rifaximin.

Effects on the inflammatory and circulatory state in patients with cirrhosis and ascites

- A randomised controlled clinical study

January 29^th^, 2015

**Eudra CT number: 2012-002890-71**

**Protocol code: RifaxNK150612**

#### Sponsor

Professor, overlæge, Dr. Med Flemming Bendtsen

Department of gastroenterology, Copenhagen University Hospital Hvidovre

Kettegaard Alle 30, 2650 Hvidovre

Tel: 38623273/ 20326836

Email: [flemming.bendtsen@regionh.dk](mailto:flemming.bendtsen@regionh.dk)

#### Principle investigator

MD, Nina Kimer,

Department of gastroenterology, Copenhagen University Hospital Hvidovre,

Kettegaard Alle 30, 2650 Hvidovre

Tel: 38623181 / 25140961

Email: [nina.kimer@regionh.dk](mailto:nina.kimer@regionh.dk) / [ninakimer@dadlnet.dk](mailto:ninakimer@dadlnet.dk)

# Table of contents

[Table of contents 2](#__RefHeading___Toc328383102)

[Primary centres of investigation 4](#__RefHeading___Toc328383103)

[Sub investigators 4](#__RefHeading___Toc328383104)

[Collaborating units 5](#__RefHeading___Toc328383105)

[Analyses and investigations 5](#__RefHeading___Toc328383106)

[Drug delivery 5](#__RefHeading___Toc328383107)

[Drug handling 5](#__RefHeading___Toc328383108)

[Surveillance and monitoring 6](#__RefHeading___Toc328383109)

[Patient screening and inclusion 6](#__RefHeading___Toc328383110)

[Time schedule 7](#__RefHeading___Toc328383111)

[Background information 7](#__RefHeading___Toc328383112)

[Aim and hypothesis 9](#__RefHeading___Toc328383113)

[Outcome measures 10](#__RefHeading___Toc328383114)

[Primary outcome measures 10](#__RefHeading___Toc328383115)

[Secondary outcome measures 10](#__RefHeading___Toc328383116)

[Study Design 11](#__RefHeading___Toc328383117)

[Trial participants 11](#__RefHeading___Toc328383118)

[Inclusion criteria 11](#__RefHeading___Toc328383119)

[Exclusion criteria 11](#__RefHeading___Toc328383120)

[Withdrawal of trial participants 12](#__RefHeading___Toc328383121)

[Dropout 12](#__RefHeading___Toc328383122)

[Follow up of possible dropouts 12](#__RefHeading___Toc328383123)

[Data registration on dropouts 13](#__RefHeading___Toc328383124)

[Disruption of the trial 13](#__RefHeading___Toc328383125)

[Recruitment of participants 13](#__RefHeading___Toc328383126)

[Recruitment of patients at primary hospitals 13](#__RefHeading___Toc328383127)

[Admission to Hvidovre Hospital 14](#__RefHeading___Toc328383128)

[Measurements and investigations 14](#__RefHeading___Toc328383129)

[Investigations on day 0 14](#__RefHeading___Toc328383130)

[Investigations on day 1 15](#__RefHeading___Toc328383131)

[Investigations on day 2 15](#__RefHeading___Toc328383132)

[Investigations on day 8 16](#__RefHeading___Toc328383133)

[Investigations on day 15 16](#__RefHeading___Toc328383134)

[Investigations on day 22 16](#__RefHeading___Toc328383135)

[Investigations on day 29-32 16](#__RefHeading___Toc328383136)

[Risks and disadvantages related to investigations 16](#__RefHeading___Toc328383137)

[Drug information 17](#__RefHeading___Toc328383138)

[Rifaximin 17](#__RefHeading___Toc328383139)

[Placebo 18](#__RefHeading___Toc328383140)

[Side effects, risks and disadvantages related to medication 18](#__RefHeading___Toc328383141)

[Possible side effects to Rifaximin 18](#__RefHeading___Toc328383142)

[Possible benefits for the trial participant 19](#__RefHeading___Toc328383143)

[Placebo 20](#__RefHeading___Toc328383144)

[Handling of trial medication 20](#__RefHeading___Toc328383145)

[Record keeping of medication 21](#__RefHeading___Toc328383146)

[Randomisation methods 21](#__RefHeading___Toc328383147)

[Disruption of blinding 22](#__RefHeading___Toc328383148)

[Establishment of a Bio bank 22](#__RefHeading___Toc328383149)

[Aim of the bio bank 23](#__RefHeading___Toc328383150)

[Plans for the bio bank 23](#__RefHeading___Toc328383151)

[Adverse events and side effects 24](#__RefHeading___Toc328383152)

[Definitions 24](#__RefHeading___Toc328383153)

[Registration of adverse events / adverse reactions 25](#__RefHeading___Toc328383154)

[Reporting of serious adverse events 25](#__RefHeading___Toc328383155)

[Reporting of Suspected Unexpected Serious Adverse reactions (SUSAR’s) 25](#__RefHeading___Toc328383156)

[Statistics 26](#__RefHeading___Toc328383157)

[Power calculation 27](#__RefHeading___Toc328383158)

[Data management 28](#__RefHeading___Toc328383159)

[Missing data 29](#__RefHeading___Toc328383160)

[Ethics 29](#__RefHeading___Toc328383161)

[Obtaining consent 29](#__RefHeading___Toc328383162)

[Patient disadvantages and risks 30](#__RefHeading___Toc328383163)

[Patient benefits 31](#__RefHeading___Toc328383164)

[Financing and insurance 31](#__RefHeading___Toc328383165)

[Insurance 31](#__RefHeading___Toc328383166)

[Finance and funding 31](#__RefHeading___Toc328383167)

[Legal assistance 32](#__RefHeading___Toc328383168)

[Publication 33](#__RefHeading___Toc328383169)

[Implications 33](#__RefHeading___Toc328383170)

[List of appendices 33](#__RefHeading___Toc328383171)

[Literature 35](#__RefHeading___Toc328383172)

# Primary centres of investigation

Gastrounit, Medical Division, Copenhagen University Hospital Hvidovre,

Kettegaard Alle 30, 2650 Hvidovre

Department of Clinical Physiology and Nuclear Medicine, Copenhagen University Hospital Hvidovre,

Kettegaard Alle 30, 2650 Hvidovre

## Sub investigators

MD, Phd, Lise Hobolth

Tel: 38621917

Email: [lise.hobolth.01@regionh.dk](mailto:lise.hobolth.01@regionh.dk)

MD, Julie Pedersen

Email: [Julie.steen.pedersen@regionh.dk](mailto:Julie.steen.pedersen@regionh.dk)

In the period april 1^st^ 2014 to January 1^st^ 2015 Julie Pedersen will be principle investigator.

Professor, overlæge dr. med. Søren Møller

Tel: 38623568

Email: soeren.moeller@regionh.dk

# Collaborating units

## Analyses and investigations

Department of clinical physiology and nuclear medicine, Copenhagen University Hospital Hvidovre,

Kettegaard Alle 30, 2650 Hvidovre

Department of clinical biochemistry, Copenhagen University Hospital Hvidovre,

Kettegaard Alle 30, 2650 Hvidovre

Statens Serum Institute, SSI, Artillerivej 4, 2300 København S

Contact: Overlæge, Andreas Munk Pedersen

Tel: 38626199 / 38625960

Email: andreas.munk.Petersen@hvh.regionh.dk

## Drug delivery

Norgine Denmark

Stamholmen 151, 6. floor

2650 Hvidovre

Denmark

Contact: John Stone MD, Medical Manager

Tel: +45 33170400

## Drug handling

Region Hovedstadens Apotek
Marielundvej 25
2730 Herlev

Contact: Områdechef ikke steril produktion Alice Rosendahl

Tel: 44 57 77 50 Fax: 44 57 77 29
Mail: [alice.rosendahl@regionh.dk](https://webmail.regionh.top.local/owa/redir.aspx?C=iWHQ3q6IS0CAtEezSgbwxYjFgRJMG88I4-zbg-vKkVio0D6_UUg7xSDkOcrR5XaPCxRXM8R3O3M.&URL=mailto%3Aalice.rosendahl@regionh.dk)

## Surveillance and monitoring

GCP Unit, Copenhagen University Hospital

Bispebjerg Hospital, Bygning 51, 3.sal, Bispebjerg Bakke 23, 2400 København NV

Telephone: 3531 3890

Name of primary contact: Jeanette Blom

Email: jeanette.blom@regionh.dk

## Patient screening

Department of Medical Gastroenterology and endocrinology, Copenhagen University Hospital Bispebjerg,

Bispebjerg Bakke 23, Indgang 60, 5. tværvej, 2400 København NV

Medical contact: Marianne Vester-Andersen: Marianne.Kajbaek.Vester-Anderen@regionh.dk

Department of internal medicine, Copenhagen University Hospital Frederiksberg

Nordre Fasanvej 57, Hovedvejen, indgang 12, 2000 Frederiksberg

Medical contact: Srdan Novovic: srdannovovic@gmail.com

Department of Hepatology, Copenhagen University Hospital Rigshospitalet

Blegdamsvej 9, 2100 København Ø

Medical contact: Ole Hamberg: ole.hamberg@rh.regionh.dk

Department of gastroenterology, Copenhagen University Hospital Herlev,

Herlev Ringvej 75, 2730 Herlev

Medical contact: Erika Belard: Erika_Belard@hotmail.com

Department of internal medicine, Copenhagen University Hospital Glostrup

Nordre Ringvej 57, 2600 Glostrup

Medical contact: Niels Johansen: niels.rene.johansen.01@regionh.dk

Department of Internal Medicine, Copenhagen University Hospital Gentofte

Niels Andersensvej 60, 2900 Hellerup

Medical contact: Eva Dahl: eva.efsen.dahl.02@regionh.dk

Department of Internal Medicine, Copenhagen University Hospital Køge

Lykkebækvej 1, 4600 Køge

Medical Contact: Sanne Dam-Larsen: [sanda@regionsjaelland.dk](mailto:sanda@regionsjaelland.dk) and Lone Madsen: logm@regionsjaelland.dk

# Time schedule

November 2012: Enrolment of the first patients and initiation of investigations.

December 2015: Enrolment of the last patients and investigations.

January 2016: End of follow up and trial lock.

Spring 2016: Assessments and analysis of first data.

Summer 2016: Publication of results.

# Background information

Ascites is a frequent complication of cirrhosis, occurring in 50% of patients within 10 years of the diagnosis and associated with 50 % mortality in two years. If hepatorenal syndrome occurs, the mean survival is only a few months. Bacterial infections in patients with cirrhosis occur at admission or during hospitalization in app. 30 % of patients(1). High Child-Pugh Score, variceal bleeding and low ascetic fluid protein levels are associated with high risk of infection(2;3).

A bacterial infection is an important precipitating event for the development of renal failure and hepatorenal syndrome, hepatic encephalopathy and possibly also variceal bleeding. The infections are mainly triggered by gut bacterial translocation, the migration of microorganisms from the intestinal lumen to mesenteric lymph nodes or extra-intestinal sites. Some studies have shown derangements in the gut micro ecology of patients with liver cirrhosis, partly due to a decrease in small intestinal motility, which could cause small intestinal bacterial overgrowth(4;5). Combined with structural and functional alterations of the intestinal mucosa and deficiencies in defence mechanisms, this bacterial overgrowth contributes to bacterial translocation.

Circulating bacterial DNA is a marker of bacterial translocation that may enhance endothelial dysfunction and predict a poor outcome in cirrhosis and ascites(6;7). Selective gut decontamination prevents spontaneous bacterial infections and improves survival in advanced cirrhosis, such as in variceal bleeding and in patients with spontaneous bacterial peritonitis (SBP)(8).

In bacterial infections due to translocation, endotoxines display their effect via TNFa-signalling and toll-like-receptor 4 (TLR4), which leads to an inflammatory response.(9-11)

This induces increased expression and activation of inducible NOS leading to excessive NO production and further vasodilatation. Patients with advanced cirrhosis are, apart from having portal hypertension, characterized by a marked systemic vasodilatation, an increased cardiac output and a low central blood volume. This could be due to raised levels of endotoxines in the blood. Furthermore, lipopolysaccharide-binding-protein (LBP), a surrogate marker of endotoxemia, as well as high levels of IL-6 and TNF-α(12;13), is associated with a lower systemic vascular resistance (SVR) and a higher cardiac output (CO) in cirrhotic patients(14;15).

In decompensated cirrhosis several vasoactive hormones are activated to counterbalance the vasodilatation.

In animal studies, it has been shown that oral antibiotics might reduce bacterial translocation and the vasodilation in the splanchnic vasculature.(16-19)

Quinolones are widely used for this purpose, yet they carry a high risk of developing bacterial resistence and risk of clostridium difficile infections[^8^](#_ENREF_8). Rifaximin seems to be an attractive alternative that exerts a broad-range of antimicrobial activity including gram-positive bacteria. Rifaximin induces less bacterial resistance and acts predominantly in the small intestine, the site of bacterial overgrowth in cirrhosis(20).

Rifaximin has been shown to reduce the risk of hepatic encephalopathy(21;22), with less side effects than traditional lactulose treatment(23). One study has implied that rifaximin might have an effect on minimal hepatic encephalopathy (24). However, evidence of the mental effect of rifaximin on covert hepatic encephalopathy is still scarce.

The hepatic venous pressure gradient (HVPG) is a marker of severity of cirrhosis and has important prognostic value in the assessment of risk of complications to cirrhosis. An uncontrolled study has suggested that rifaximin may decrease HVPG(8). A recently published pilot study has furthermore suggested, that rifaximin might improve systemic hemodynamics by decreasing cardiac output and also increasing glomerular filtration rate (GFR)(25).

The overall idea of this trial is to assess the impact of rifaximin on the flow of events as described in the figure below, by blocking the second hit bacterial overgrowth and translocation and thereby improving splanchnic and systemic haemodynamics.

This novel, investigational trial will be assessing the effect of rifaximin on pathophysiology and haemodynamics in the patient with liver cirrhosis, and addressing several organs on marker level.

We will explore the molecular and physiological effects of rifaximin rather than confirming evidence of its already known beneficial effects.


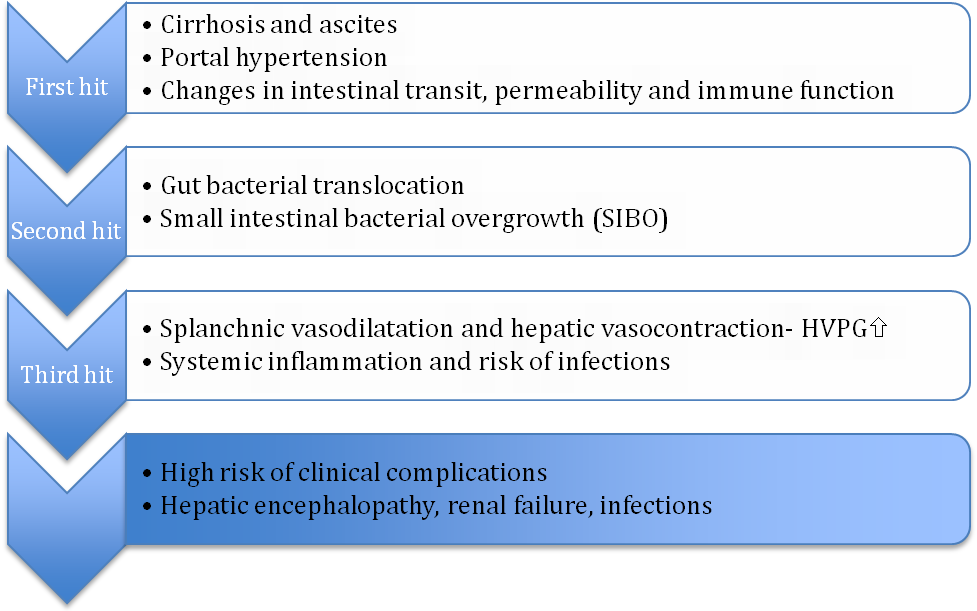


# Aim and hypothesis

We hypothesize that intestinal decontamination with rifaximin in patients with cirrhosis and ascites will interrupt the progression of the disease from the first to the second hit as described in the figure. Based on these assumptions, we hypothesize, that rifaximin may have the following effects:

1. Will decrease portal pressure, measured as the hepatic venous pressure gradient (HVPG).
2. Will ameliorate the peripheral and splanchnic vasodilatation by a decrease in cardiac output (CO) and an increase in arterial blood pressure and systemic vascular resistance (SVR). These effects should also be reflected by a trend towards normalisation of vasoactive hormones.
3. Will improve renal function expressed as an increase in glomerular filtration rate.
4. Will downregulate markers of inflammation expressed as a decrease in proinflammatory cytokines (i.e. TNF-α and interleukines) and high sensitivity CRP.
5. Attenuate markers of infection, expressed by bacterial DNA and lipopolysaccharide binding protein (LPS-BP).

## Outcome measures

The aim of this trial is mainly explorative and investigational and we aim to address a series of haemodynamic and biochemical parameters as to assess the effect of rifaximin comprehensively. Hence, we have included a range of outcome measures that will be addressed individually and in general.

## Primary outcome measures

Hepatic venous pressure gradient (HVPG), measured as the gradient between wedged and free hepatic venous pressure.

Glomerular Filtration Rate (GFR) assessed by chrome-EDTA clearance

## Secondary outcome measures

Cardiac Output (CO), assessed by indicator dilution technique.

Systemic vascular resistance (SVR) measured as dynxsxcm-5,

Mean arterial blood pressure (MAP) measured as mmHg,

Heart Rate (P) measured as beats/minute,

Biochemical markers of bacterial translocation and proinflammatory cytokines,

Effect on vasoactive hormones

Improvement in minimal Hepatic encephalopathy

Effect on weight and need of diuretics

Hospitalization during the treatment period,

Infections during the treatment period

Mortality and co morbidity during a follow-up period of 6 months.

# Study Design

## Trial participants

Our trial population will consist of patients with alcohol-related liver cirrhosis Child-Pugh score B or C and ascites, who in one of the involved departments are planned to be offered a liver vein catheterisation for assessment of hepatic venous pressure gradient (HVPG), according to local guidelines.

The sub investigator at the referring centre is a trained physician that handles the patient’s daily treatment. The sub investigator will identify patients in need of liver vein catheterisation and for whom it could be relevant to participate in the trial and who meet inclusion criteria.

Both newly referred patients and patients with a history of cirrhosis will be offered inclusion in the study.

## Inclusion criteria

- Patients with decompensated liver cirrhosis and ascites, verified by ultrasound or CT scan within the last 3 months
- Age between 18 and 80 years.
- Portal hypertension and a hepatic venous pressure gradient (HVPG) of 10 mmHg or more.
- Women of child-bearing age should use safe anti conception, defined as either hormonal anti conception or intrauterine device.

## Exclusion criteria

- Child-Pugh Score above 13
- Clinical signs of infection evaluated by blood biochemistry, urine culture and if applicable ascites puncture, and through clinical assessment by the investigator.
- Received antibiotic treatment within 14 days prior to inclusion
- Presence of hepatocellular carcinoma
- Ongoing invasive cancer or invasive cancer within the last five years,
- Overt hepatic encephalopathy (HE above grade 1),
- Serum creatinine > 200 μmol/l,
- Transfusion requiring bleeding within one week prior to inclusion,
- Severe cardiac, pulmonary or kidney diseases or Type 1 diabetes mellitus,
- Continuous alcohol abuse with symptoms of abstinences
- Expected survival less than 3 months,
- Denied consent
- Plasma haemoglobin of less than 5,5 mmol/L

## Withdrawal of trial participants

Trial participants are withdrawn from the study if they:

- Are in need of other antibiotics during study period,
- Are admitted to hospital for other morbidity and require treatment that is incompatible with continuous rifaximin treatment.
- If blinding is repealed.
- Experience deterioration in hepatic encephalopathy above grade 1 or develops hepatic encephalopathy, which will prevent further participation in the trial.

## Dropout

A trial participant will be classified as dropped out of the study if one the following criteria are met:

- The trial participant has ingested less than 32 tablets of rifaximin 550 mg or placebo, equal to 16 days of treatment. That is, less than rifaximin 17.600 mg or placebo, equal to 57% of planned treatment.
- The trial participant withdraws his or hers written consent.

## Follow up of possible dropouts

**Trial participants that fail to appear for weekly controls or admission to hospitals according to study design will be sought contacted by email, telephone and at their home address in that given order. If a participant is impossible to reach in this manner, he or she will be considered as dropped out of the study. Further medication will be withheld and the participants primary hospital will be informed, so as to undertake his or hers regular medication and controls.**

**We have not planned any follow-up or additional treatment for dropouts.**

## Data registration on dropouts

The following data on dropouts will be sought registered:

- Reason for drop out
- Morbidity explaining drop out
- Mortality
- All patient data collected for the time period the trial participant were included in the study, unless the patient withdraws his or hers written consent and solicits eradication of data.
- Amount of ingested Rifaximin/placebo and amount of remaining extradited Rifaximin/placebo.

## Disruption of the trial

The entire trial will be disrupted prematurely if knowledge is obtained that suggests rifaximin is unsafe or indefensible to use in patients with liver cirrhosis.

# Recruitment of participants

## Recruitment of patients at primary hospitals

The attending physician in one of the referring centres will identify patients that are eligible for the trial.

The patient will on a regular outpatient control receive oral information about the trial and be offered inclusion. Trial information and written consent is handed out. On the basis of this information the patient can choose to give his or hers written consent to participation in the trial.

The attending physician will offer the patient an information session held by the principle investigator in the outpatient clinic of the patient’s primary hospital. At this session written consent form must be signed.

It must be underlined, that all relevant procedures for diagnosing and treating the patient’s liver cirrhosis will be completed according to hospital guidelines and best practice irrespective of the patient’s inclusion in the trial.

### Referral to Hvidovre Hospital

The attending physician refers the patient to Hvidovre hospital for liver vein catheterization, with a note of trial inclusion.

The patient is hereafter referred to as the trial participant. The trial participant will receive a set of written information about procedures and trial course by mail or by the principle investigator before admission to hospital.

## Admission to Hvidovre Hospital

On the admission date, day 0 in the following, the principle investigator or a sub investigator registers the patient. Information about the trial is supplemented if necessary and written consent is verified.

# Measurements and investigations

The following flow-chart for the trial is applied. An extensive list of investigations and blood samples are given in appendix 6.

All scheduled investigations in this trial are standard procedures at the department of clinical physiology and nuclear medicine, Hvidovre Hospital. As well as blood analyses are standard procedures at the department of clinical biochemistry, Hvidovre hospital.

Standard Operating Procedures for all included investigations are therefore available to study nurses, investigators and other personnel participating in the survey and treatment of participants on the intranet for Hvidovre Hospital(26).

## Investigations on day 0

- The trial participant is fasting at admission to the hospital.
- Glucose breath test is performed to assess bacterial overgrowth.
- Hospitalisation and registration of the trial participant.
- Clinical examination by investigator or sub investigator and registration of clinical signs symptoms and basic values, That is height, weight, blood pressure, pulse, temperature.
- Routine blood samples for evaluation of kidney and liver function and possible infection (specified in appendix 6)
- To exclude occult infection: Blood culture and urinary culture.
- All women of childbearing age will have taken urine-HCG to exclude pregnancy.
- Diagnostic ascites puncture to exclude bacterial peritonitis, in case the patient has clinical ascites.
- Samples of faeces to assess bacterial composition in the gut.
- Examination of continuous reaction time (CRT) to assess possible minimal hepatic encephalopathy.
- Psychometric Hepatic Encephalopathy Score (PSE score)

All of the above blood analyses are done on peripheral blood, in total 65 ml of blood, and can be withdrawn from the same puncture site, except blood culture.

## Investigations on day 1

- The participant is fasting from the morning.
- DEXA scan is performed to determine body composition of fat, fat free mass and bone mineral composition.
- Liver vein catheterisation is performed including measurements of splanchnic haemodynamics and metabolism and systemic haemodynamics (analyses specified in appendix 6).
- Blood samples are drawn during catheterisation.
- Assessment of renal function (Cr-51-EDTA-clearance for determination of glomerular filtration rate, GFR)
- Measurements of biomarkers of renal function (specified in appendix 6)
- Samples of blood will be drawn for analysis of vasoactive hormones, cytokines, and markers of infection. (Specified in appendix 6)
- Hereafter, the participant will enter randomisation.

All blood analyses are done on liver vein blood or arterial blood from the femoral artery, in total 120 ml. Analyses for glomerular filtration rate are done on peripheral blood, this is included in the 120 ml.

## Investigations on day 2

- The first dose of active drug/placebo is given and the patients receive medication for 2 weeks.
- The participant will receive both oral and written information about how to administer the medication. The participant is discharged with a scheduled clinical control two weeks from this date.

## Investigations on day 7-10

- The patient is contacted by telephone by an investigator.
- Information on medicine ingestion and compliance is registered.
- Possible side effects, complications and wellbeing are also registered according to case report form.

## Investigations on day 14-17

- All participants are scheduled for clinical control in the outpatient clinic by an investigator.
- At the visit eventual unused tablets are counted, the patient is interviewed on compliance to treatment and ingestion of medication. Medication for the remaining trial period is handed out.
- Side effects, complications and wellbeing are also registered according to case report form.

## Investigations on day 21-24

- The patient is contacted by telephone by the study nurse or an investigator
- Information on medicine ingestion and compliance is registered.
- Side effects, complications and wellbeing are also registered according to case report form.

## Investigations on day 29-32

- At the end of the trial period, patients are re-hospitalized
- Programs of day 0 and day 1 are repeated, elaborated in appendix 6.
- Blood culture and urine culture are avoided if the participants show no signs of infection.
- A three-day treatment window is allowed to avoid admission of patients during weekends and holidays.

All blood analyses are done on liver vein blood, arterial or peripheral blood as specified above, in total 165 ml.

## Follow up

A register based follow up on patients through medical records will be performed at the end of trial, which is 1 month after last patient’s last visit. Data collection will be limited to hospital admissions and reasons hereto, complications to liver cirrhosis (esophageal bleeding, spontaneous bacterial peritonitis, hepatic encephalopathy and hepatorenal syndrome), and mortality and reasons hereto.

Hence, patients will be followed up until death, or end of trial.

# Risks and disadvantages related to investigations

Liver vein catheterisation and routine blood samples are part of the regular control and investigations of patients with liver cirrhosis. Procurement of extra blood and blood samples will be taken in addition to the patient’s admission to hospital for liver vein catheterisation. This is to avoid unnecessary blood sampling.

The study design requires the patients have all investigations repeated after 28 days to assess the effect of rifaximin. This necessitates a new admission to hospital for a minimum of 48 hours.

During blood sampling there is a small risk of developing haematoma at the puncture site. For liver vein catheterisation there is a small risk of developing haematoma at the puncture site or the femoral vein and artery. In rare cases the patient can develop vasovagal reaction during the investigation, this will be treated with an intravenous injection of atropine. Seldom the patient can develop an allergic reaction to the local anaesthetic used at the puncture site.

There are no known side effects related to chrome EDTA clearance or to glucose breath test.

Prior to liver vein catheterisation and glucose breath test the patient must be fasting for 8 hours. This can lead to mild nuisances such as thirst, headaches and dizziness. This will be sought remedied with intravenous fluids.

A DEXA scan of the body will expose the patient to a relatively small dose of radiation in the range of 0,001 to 0,01 mSv (27). In relation to investigation of glomerular filtration rate and central blood volume a small amount of radioactive isotopes will be injected in the patient, equivalent to an efficient radiation dose of 0,006 mSv and 1,8 mSv, respectively (27). For comparison background radiation in Denmark is 2-20 mSv pr. year.

# Drug information

This clinical trial will compare the antibiotic tablet rifaximin in the dosage 1100 mg daily for 28 days to tablet placebo.

Both rifaximin and placebo tablets will be delivered from Norgine Denmark free of charge. The sale name of rifaximin is Xifaxan. Each tablet of Rifaximin contains 550 mg of rifaximin. The tablets of placebo will be similar in size, shape and weight as the Rifaximin tablet.

## Rifaximin

Rifaximin, an antibacterial drug that is poorly absorbed from the gut, belongs to the group of rifamycins. Rifaximin has wide antibacterial range acting against most gram positive and negative, aerobic and anaerobic bacteria in the gut. Rifaximin acts locally in the gastrointestinal lumen, and has no clinical effect on invasive bacteria. Less than 1 % of the drug is absorbed, and the risk of interaction with other drugs is very little. Also excretion of rifaximin in urine is less than 0,4 % of administered dose.

Rifaximin treatment prevents bacterial overgrowth in the small intestine(20), thereby possibly preventing gastrointestinal infections and bacterial translocation to organs and bloodstream. Moreover, we expect the prevention of bacterial translocation to have a positive effect on the haemodynamics by decreasing hepatic venous pressure and increasing systemic blood pressure. This will be elaborated in the following.

Furthermore, rifaximin has been shown to reduce the risk of hepatic encephalopathy(21), a common complication to cirrhosis, and is approved by the FDA (U.S. Food and Drug administration) for treatment and prevention of hepatic encephalopathy.

In Denmark, rifaximin is currently approved for the treatment of travelling diarrhoea caused by non-invasive gut bacteria. Recommended dosage is 200 mg thrice daily for three days(28).

The aim of increased dosage and prolonged treatment (1100 mg daily for 4 weeks, as elaborated in the following) is to reflect the recommended dosage used for hepatic encephalopathy in USA, which has proven to be efficacious in this group of patients, when suffering from hepatic encephalopathy. Moreover, radical eradication diminishes translocation of bacteria and bacterial DNA across the gut mucosa and in to the blood stream. Hepatic venous pressure, systemic blood pressure and hepatic encephalopathy are complex modes and we expect prolonged treatment to be necessary to detect changes and achieve significant results. In previous trials of Rifaximin for the treatment of hepatic encephalopathy and rifaximin for intestinal decontamination, a daily dose of 1200 mg is administered.(8;29)

## Placebo

Placebo tablets are in appearance identical to the rifaximin tablets, but contain no active substances. A description of placebo tablets is found in appendix 10.

# Side effects, risks and disadvantages related to medication

## Possible side effects to Rifaximin

The tolerability of rifaximin has been evaluated in approximately 1000 patients in 30 clinical trials(30). Very few adverse events were reported during short-term treatment, the most common being gastrointestinal in origin, (fatigue, nausea, dizziness, pruritus and abdominal pain). It is worth noticing that these symptoms are in some ways similar to those of the patient’s underlying disease, and adverse events could have been underreported.

Prolonged treatment with high doses of rifaximin has been associated with urticarial skin reactions.

However, label information regarding rifaximin (Xifaxan) for the American market reports adverse events at either the same frequency in both rifaximin and placebo groups (31;32), or at a higher frequency in the rifaximin group (32). These reactions occur with an incidence of > 5% and relates to: peripheral oedema, nausea, dizziness, fatigue, ascites, muscle spasms, abdominal pain, anaemia, insomnia, upper abdominal pain, back pain, constipation, pyrexia, dyspnoea, nasopharyngitis and rash. Once again these symptoms are similar to the unspecific symptoms of the patient with liver cirrhosis.

In the reviewing of rifaximin for approval for treatment of hepatic encephalopathy in FDA clinical trials comprising 757 patients were evaluated(31). The adverse event profile matched that of placebo.

There is no available data on the use of rifaximin in patients with decreased kidney function. In patients with impaired liver function, no particular adjustments in dose are recommended. Data on rifaximin and its impacts on pregnancy and breastfeeding are scarce, and usage is deprecated(32).

Rare side effects (> 1/1000 to <1/100) such as increased transaminases, muscle cramps and muscle weakness, upper respiratory tract infections and increased blood pressure do appear(28).

## Possible benefits for the trial participant

Rifaximin has been shown to reduce the incidence of spontaneous bacterial peritonitis (SBP) and correct small intestinal bacterial overgrowth (SIBO) in patients with alcoholic liver cirrhosis(33;34). Intestinal decontamination may also demonstrate an improvement in liver haemodynamics(8), systemic haemodynamics and renal function(35).

These effects may contribute to the overall well-being of the patient and prevent complications to the underlying cirrhosis such as risk of infections, progression of disease, and admission to hospital.

It has also been certified that rifaximin reduces the risk of overt hepatic encephalopathy episodes(21), and improves quality of life and psychometric performance in these patients(24;36). Moreover, hospitalisation associated to HE can be reduced(31).

It is possible, but not certain, that the participants could experience some of these benefits.

This is a pathophysiological and investigational trial, and besides the mentioned benefits for the participant, he or she will also contribute to the possible development of new treatment regimens for liver cirrhosis.

## Placebo

Placebo medication is described and outlined in Investigational Medicines Product Dossier in appendix 10. We expect no benefits or disadvantages, nor risks or side effects related to placebo medication.

# Handling of trial medication

The patients will receive 550 mg of rifaximin twice daily for 28- 32 days in total. That is 1 tablet of 550 mg two times daily. The patients randomized for placebo will receive the equivalent amount of placebo tablets in the same intervals.

Treatment dose is justified in the section Drug information above, p. 17.

The medication is an oral tablet that can be ingested with a glass of water. Dosage is two times daily, morning and evening.

The regional pharmacy will pack medication in two containers for each participant, the first container comprising 28 tablets for the first 14 days of treatment and the second container comprising 34 tablets for the remaining 14-18 days of treatment.

The regional pharmacy will label medication and placebo containers according to GMP guidelines for labelling of medication for clinical trials.

All containers for the last treatment period will contain medication for 18 days, hence the 36 tablets.

At the end of the study participants will be readmitted to hospital, and due to normal workflow and weekends we cannot guarantee readmission to hospital on day 28 for all.

Therefore we have allowed a treatment window of four days at the end of the trial.

Rifaximin tablets and placebo will be similar in size, colour, shape and form.

## Record keeping of medication

The primary investigator, a sub investigator or a study nurse will keep record of medication and hand out medication when participants are present for the two-weekly control. Registration of medicine delivered from pharmacy, handed out to participants and unused medicine returned by participants will be executed.

Patients will be informed both orally and in writing to return all unused medication to the hospital and investigators. The primary investigator and study nurse will register used and unused medication and keep track of the ingested dose of each participant. The participant will also be asked to keep a journal of medicine intake. In cases where the amount of ingested medication is unclear, the highest certain dose ingested will be registered. In cases of lost medication or if the participant is unable to account for the medication, the ingested dose will be registered as null.

# Randomisation methods

The study is conducted as a randomised placebo controlled clinical trial, randomised in the ratio 2:1, rifaximin and placebo, respectively.

We expect no changes in clinical state, haemodynamics or other parameters in the patients receiving placebo, therefore we find it adequate to randomize in the ratio 2:1.

The study is dimensioned at including 32 patients in the rifaximin group and 16 patients in the placebo group.

The trial is a multi centre study, with seven hospital departments screening possible candidates for participation and referral to primary centre of investigation.

Packing and labelling will take place at the regional hospital pharmacy. Labelling will be performed after applicable legislation including name of pharmacy, trial name, patient name, CPR and trial number, dose, storage and contact information of principle investigator.

The randomization list in the ratio 2:1 will be produced electronically. This randomization list will be stored at the regional pharmacy. Only personnel at the pharmacy will know the randomisation code.

Participants will be assigned to a randomisation number electronically.

This two-digit trial number will henceforth identify trial participants.

The sponsor, principle investigator and sub investigators, nurses, laboratory assistants or personnel involved in the care for the trial participant will have no knowledge of the blinding. Coded envelopes with randomisation keys to each participant will be kept at Hvidovre Hospital in a safe and locked drawer. These envelopes will be available to sponsor and investigators at all times in case unblinding should be necessary.

## Disruption of blinding

Blinding will be disrupted if one of the following events occurs:

- A trial participant is admitted to hospital with a severe or life-threatening condition where rifaximin is contraindicated.
- A trial participant experiences serious adverse side effects or complications that could be caused by rifaximin.

If blinding is disrupted, the patient will be excluded from the trial.

# Establishment of a Bio bank

In connection to this study a research bio bank and a bio bank for future research will be established. The objective is that the bio banks will contain material from participants before and after trial medication. The material is full blood, EDTA plasma, urine, ascites and faeces samples.

In connection with liver vein catheterisation an amount of blood will be drawn for storage in a research bio bank, located at Department of clinical physiology and nuclear medicine, Hvidovre hospital.

The blood will be drawn during liver vein catheterisation from the same tube as the remaining blood samples and implies no further risks, side effects or nuisances to the patient. Urine samples are taken from the collection of 24-hour urine. Ascites is taken by diagnostic puncture.

The blood, urine and ascites is immediately preserved at a temperature of –80 degrees Celsius. These samples are stored at department of clinical physiology and nuclear medicine, Hvidovre Hospital.

Faeces samples from all patients will be stored at a temperature of –80 degrees Celsius at Statens Serum Institute.

## Aim of the bio bank

The established bio bank will have two sections and serve two purposes:

The research bio bank will contain blood samples that for various reasons are not ready for immediate analysis. These samples will be stored in a research bio bank during the trial course and analyzed for results during the trial period. This accounts for app. 10 ml of full blood. The blood will be used for cytokine analysis, bacterial DNA analysis and analysis for vasoactive hormones. Data from these samples will enter the database and registers of this trial, and excess blood from these analyses will be destroyed hereafter. This material is person attributable.

Second, a bio bank of full blood, plasma, urine, ascites and faecal samples for future research will be established. The material accounts for 30 ml of full blood at initiation and at the end of trial period and in total 20 ml of urine, 20 ml of ascites and 2 faecal samples. A part of this blood will undergo analyses for cytokines and bacterial DNA; the remaining blood, urine, ascites and faeces is stored for investigations that are not yet planned or initiated.

The aim of this bio bank is to contribute significantly to future research and thereby optimize treatment of liver cirrhosis. This material is person attributable.

All investigations and results derived from the bio bank for future research will serve the purpose of improving the life and health of patients with liver cirrhosis.

Permission from The Danish Data Protection Agency, Medical Products Agency and regional Science Ethics Committee will be obtained before initiation of this trial.

## Plans for the bio bank

Blood samples stored for later investigation in relation to this trial will be destroyed at the end of the trial. The primary investigator will store results of analyses until statistical processing. Storage of data, trial material and CRF’s will be kept in accordance to general guidelines for storage of person attributable material, set by The Danish Data Protection Agency.

The remaining blood and material stored for future research in the bio bank will be person attributable as well. No material stored in the bio bank will be exported to other hospitals, research facilities or to other countries.

When or if the material concludes in another study and future investigations, a new protocol and repeated registration at Data Protection Agency will be performed.

The blood will be stored for a period of 10 years after the end of this trial. Then the blood will be destroyed.

# Adverse events and side effects

## Definitions

An adverse event is: Any untoward medical occurrence in a patient or clinical trial subject administered a medicinal product and which does not necessarily have a causal relationship with this treatment.

A serious adverse event is: Any untoward medical occurrence or effect that at any dose results in death, is life-threatening, requires hospitalisation or prolongation of existing hospitalisation, results in persistent or significant disability or incapacity, or is a congenital anomaly or birth defect.

An adverse reaction is: Any harmful and unwanted reaction towards a trial drug irrespective of dose.

An unexpected adverse reaction is: Any harmful and unwanted reaction towards a trial drug, whose character or severity does not correlate to product information (investigators brochure).

These definitions regards to medical treatment, investigations and invasive procedures performed during the trial.

Events and reactions that will not be handled as serious adverse events:

- Adverse events and reactions that with certainty are occurring in relation to or are caused by the treatment and diagnosis of other medical conditions or diseases besides liver cirrhosis during the trial period will not be reported.
- Planned admission to hospital due to diagnostic procedures.
- Medical conditions that can be expected as complications to the underlying progressive liver cirrhosis will not be considered adverse events, serious adverse events or unexpected adverse reactions. That is, episodes of hepatic encephalopathy, variceal bleeding, hyponatriemia, SBP and infections related to liver cirrhosis, and hospitalisation due to any of these causes.
- Abnormal blood analyses that is regularly seen in patients with liver cirrhosis, such as a fall in albumin or platelet count or an increase in INR, P-bilirubin or P-ammonium levels.

In any questions of doubt regarding the above, investigators will inform sponsor and an assessment of possible causality will be performed.

## Registration of adverse events / adverse reactions

Investigator and sponsor has in the enclosed statement obliged to comply with protocol, including to report all adverse events, serious adverse events and suspected unexpected serious adverse reaction to relevant authorities as outlined by the Danish Health and Medicines Authority(37) and The European Commission(38).

AE and SAE’s: At every contact, both by telephone and in person, the participant will be asked if adverse events have occurred since the last contact. All adverse events is registered in the Case report Form. Serious adverse events are also registered on the SAE form included in Trial Master File. All adverse events will be followed until stabilisation, termination or decision of causality.

## Reporting of serious adverse events

Information on all serious adverse events is registered in the SAE form included in Trial Master File. All serious adverse events is reported to principle investigator (Nina Kimer) or Sponsor (Flemming Bendtsen) no later than first coming workday after investigator has become aware of the event. The immediate report will be followed by a detailed written report within 24 hours. Adverse reactions, that are not life threatening and mild in character, do not require medical attention or intervention and are insignificant to the patients’ further involvement in this trial will not be reported to sponsor. These will be registered in case report forms and available to monitoring units and sponsor at any time.

## Reporting of Suspected Unexpected Serious Adverse reactions (SUSAR’s)

Any serious adverse event that is assessed to have causal relation to the trial drug is a serious reaction. Unexpected serious reactions (not previously described in relevant product information(32)) are registered as SUSAR’s.

Sponsor will gather all relevant information about the SUSAR’s. In case of death or life threatening reactions The Danish Health and Medicines Agency will be informed within 7 days of sponsor’s knowledge of the reaction.

Within 8 days a report of sponsor and investigators follow-up on the incident will be forwarded. Reporting will be done electronically.

All other SUSAR’s will reported to The Danish Health and Medicines Agency within 15 workdays.

In case of a SUSAR sponsor and principle investigator will assess the consequences and impact of the event before unblinding. Hereafter unblinding will be performed before reporting to authorities. In the evaluation of possible SUSAR’s investigators brochure for Xifaxan 550 mg (provided by Norgine Denmark, see appendix) will be addressed.

# Statistics

Statistics are based on an assessment of demography and incidence of liver cirrhosis Child-Pugh B and C in the capital region of Denmark, which will be the inclusion area of patients.

Taking exclusion criteria in to consideration, we have estimated that a realistic inclusion figure will be between 60 and 70 patients. In this group of patients we expect a drop out rate of 20-25 %.

We expect to achieve a reduction in HVPG as well as an increase in glomerular filtration rate (GFR). The smallest clinically relevant difference in measurements are set to 20 %; that is, a 20% reduction in HVPG and a 20% increase in GFR.

Data will be analyzed paired and independently (as changes between individuals and difference between groups).

Parametric and non-parametric analysis will be used, depending on the underlying distribution.

The following power calculation is based on Student t-test, with a paired testing, and a fixed sample size of 32 treated patients.

Evident information on mean values of GFR and HVPG is inconclusive. The following analyses are made on available sources(39;40) and our best estimate.

In consideration of the relatively small population size, we plan no interim analyses.

Therefore, including a dropout rate of minimum 20-25 %, our aim is to include 32 + 6 = 38 patients for treatment and 16 + 3 = 19 patients for placebo. Hence, the following power calculations are done with a fixed sample size of 48 patients, 32 in treatment group, 16 in placebo group.

The trial will be disrupted when we have included enough patients to meet sample size and power.

We plan to perform both intention to treat and pr. Protocol analyses. Hence, all patient data available will be incorporated into analyses.

## Power calculation

Power calculation is performed using the free software PS Power and Sample Size Calculations version 3.0.43(41).

### Paired analysis:

We expect a 20 % reduction in HVPG.

Mean HVPG in patients with liver cirrhosis: 17,9 mmHg(39)

DIFF: (17,9 x 0,2) ~3,58 mmHg

Probability of falsely rejecting the null-hypothesis (type 1 error): 0,05 (95% CI)

Standard Deviation equal to 95% of readings: 3,9 mmHg

The paired comparison including 32 rifaximin treated patients will have a power of **0,998** expecting a 20 % (3,1 mmHG) reduction in HVPG.

We expect a 20 % increase in GFR.

Mean GFR in patients with liver cirrhosis and non-refractory ascites: 69 mL/minute(40)

DIFF: (69x 0,2) ~13,8 ml/minute

Probability of falsely rejecting the null-hypothesis (type 1 error): 0,05 (95% CI)

Standard Deviation equal to 95% of readings: 16 ml/minute

The paired comparison including 32 rifaximin treated patients will have a power of **0,996** expecting a 20 % reduction in GFR.

### Independent analysis:

We expect a 20 % reduction in HVPG. Same values as for paired analysis are applied.

Relation between treated and controls: 2:1

We have 32 experimental patients and 16 patients receiving placebo. The HVPG within the subject group was normally distributed with standard deviation 3,9 mmHg. If the true difference in the experimental and control means is 3,1 mmHg, we will be able to reject the null hypothesis that the population means of the experimental and control groups are equal with probability (power) **0,835**.

We expect a 20 % increase in GFR. Same values as for paired analysis are applied.

Relation between treated and controls: 2:1

We have 32 experimental patients and 16 patients receiving placebo. The GFR within the subject group was normally distributed with standard deviation 16 ml/minute. If the true difference in the experimental and control means is 13,8 ml/minute, we will be able to reject the null hypothesis that the population means of the experimental and control groups are equal with probability (power) **0,79**.

We do not expect any changes in patients receiving placebo on any of the outcome measures.

# Data management

Data in this trial is retrieved from the patient’s medical records, the patient’s oral statements, laboratory results regarding biochemistry and investigational results. All data regarding this trial will be stored and kept in the electronic case report form that will function as an encrypted database. All data is handled confidential. The CPR number and the two-digit trial number identify patients. Laboratory results and investigational results are manually transferred to the electronic CRF to collect all study data in one database. The entry of data is performed by investigators or study nurse.

This makes it possible to extract data and continue or repeat data analyses if relevant or necessary in the future.

The electronic case report form and database is accessed electronically with personal user ID and password.

Only the principle investigator and sub investigators handling the care and registration of participants and situated at Hvidovre hospital will have encoded access to the electronic CRF and the encrypted database.

At the end of the inclusion period data will be extracted and analysed by primary investigator. Primary analysis will address the primary outcomes of this trial; thereafter analysis of secondary outcome measures will be performed. Data that are collected, but not immediately analysed is stored anonymously and may conclude in later analyses and statements. We aim at publishing all results derived from this trial.

## Missing data

During the trial period missing data is initially sought in the patient’s medical record. If the parameter is not available at the time of primary analysis the registration will be left empty. Primary investigator and sub investigators will pursue the options of repeating biochemistry and blood analyses when this is possible to complete data. Invasive procedures will not be repeated, should data be missing after the patient has completed the trial investigations.

At the end of the trial, all data will be published in an anonymous form.

# Ethics

This study will be conducted according to national legislation on health and ICH-GCP guidelines as mediated by the GCP unit, Copenhagen University Hospital.

The GCP-unit, Copenhagen University Hospital will monitor all aspects of the trial according to GCP guidelines.

The trial will be reported to the Regional Science Ethics Committee of the capital region of Denmark.

Permission from The Danish Data Protection Agency and Danish Medicines Agency will be obtained before initiation of this trial.

Authorities will have full access to data, documents and registration procedures during monitoring, audits and inspections.

## Obtaining consent

Participants will only be recruited through regular outpatient clinics in the capital region of Denmark and performed by the patient’s regular physician. Advertisement or recruitment will not be performed elsewhere.

The patient’s regular physician will inform patients in oral at the patient’s primary hospital.

If the patient indicates interest in the trial, written participant information is handed out (appendix 1,2 and 3). The patient will also be informed that he or she is in title to reflection and can request an extra interview where he or she can be informed in oral. All information will be provided in private consultation rooms, unless the patient wants it elsewhere. Patients with liver cirrhosis are chronically ill and spend some time on outpatient visits and controls. The information procedure is chosen to avoid contacting the patients further and to secure a safe and familiar environment for interview and information.

Contact information on the principle investigator will be provided in oral and in writing. During admission participant information, patients’ rights regarding participation in medical trials and a resume of the protocol in Danish will be available at all times to the patient.

The patient can choose to sign the written consent form at the outpatient clinic with his or hers primary physician.

When written consent is obtained the patient will be enrolled in the trial and referred to Hvidovre Hospital. Written consent forms will be forwarded to principle investigator by fax, in scanned version on email or by registered mail, to ensure that they are in hand when the participant initiates the trial at Hvidovre.

## Patient disadvantages and risks

Participants randomised to rifaximin treatment may experience side effects to medication; Risks and side effects related to medication and placebo are elaborated on p. 18.

All investigations are thoroughly described in appendix 6. Risks and complications related to investigations are elaborated on pp. 14-16.

Participation in this trial requires more than two days extra hospitalisation and an extra outpatient visit, during the 4 weeks of trial course. Moreover, we repeat a whole series of investigations, not all relevant or beneficial for the individual patient at the time of investigation. There are some nuisances and pain related to the investigations, even though we will try to compensate and relieve the participants as much as possible. The patients will, irrespective of their participation in this trial be offered elucidation of their liver cirrhosis with liver vein catheterisation, and we have placed the initial investigations in relation to this admission to hospital, hence to minimize days of hospitalisation.

One third of participants will receive placebo medicine. At present, there is no registered medication, that prevents bacterial translocation from the gut and actively lowers the hepatic venous pressure, and it lacks yet to be proven whether rifaximin has an effect on these parameters. Hence, patients are not withheld proved active medication. All patients continue their regular prescribed medication during the trial period.

Participants randomised to placebo treatment will most likely not experience any differences or improvement in symptoms and events related to their disease.

The use of placebo is considered necessary to secure that observed effects are ascribed to rifaximin with certainty.

## Patient benefits

Participation in this trial contributes to new knowledge about the foundations and scientific background that causes the serious complications to liver cirrhosis, thus enabling us to improve diagnosis and treatment of this disease and its co morbidity.

At the same time participation helps in understanding the possible mechanisms and possibilities of rifaximin in prevention and treatment of liver cirrhosis and its complications.

It is possible that trial participants randomised to rifaximin treatment will experience an improvement in both kidney function and bowel function, with less distress, flatulence or diarrhoea. It is also possible that this group of patients will have fewer complications to their cirrhosis, such as fewer admissions to hospital, fewer infections and a smaller risk of variceal bleeding and SBP.

It is also a possibility, but not certain, that participants randomised to rifaximin will experience improvement in their mental status and their general condition, for example less fatigue, better appetite and more strength.

Overall, we have evaluated benefits and risks of this trial and assessed it to be ethically secure.

# Financing and insurance

## Insurance

Patients participating in this trial will be insured through the national patient insurance. Trial participants are informed of this relation in written information of trial participant’s right, appendix 2. Sponsor and investigators are covered by Hvidovre Hospital’s statutory insurance.

Norgine Denmark is covered by their own product liability insurance that covered faults in medicine.

## Finance and funding

Rifaximin tablets of 550 mg and placebo tablets in the amounts 2520 tablets (180 blister of 14) and 1260 tablets (90 blisters of 14) respectively, equal to treatment of 38 patients with rifaximin and 19 patients with placebo, are delivered by Norgine Denmark free of costs. See appendix for contracts and agreements.

Norgine Denmark is not further involved in the trial and has no influence whatsoever on the design and methods, nor on compilation and publication of results. For business contract see appendix 7.

Norgine Denmark has not provided funding in addition to the above.

Sponsor and principle investigator have invented the overall template of this research project and have taken initiative to execute it. None of the involved researchers have economic interests or affiliations in private firms, funds or similar.

Research results obtained from this trial are of scientific interest entirely. There are no economic or commercial conflicts regarding the investigations.

The salary of primary investigator is partly financed through employment at Department of clinical physiology and nuclear medicine, Hvidovre Hospital and partly through scholarships and funds.

The department of gastroenterology Hvidovre Hospital will undertake expenses regarding extra outpatient visits and admission to hospital for the purpose of participating in this research project.

Additional costs and expenses will be financed through funds. Expenses regarding analyses and investigations will be covered through funds.

At present we have received grants from The medical foundation of Hvidovre Hospital for the treatment of liver diseases (122.000 kr. + 150.000 kr.) and the Research Foundation for the Capital Region of Denmark (1.000.000 kr.), The Research Foundation Hvidovre Hospital (50.000 kr.), Novo Nordisk Foundation (300.000 kr.), Aase og Ejnar Danielsens Fond (200.000 kr.) and Inge og Per Refshalls Fond (32.400 kr.).

Contributions and scholarships are placed on a research account administered by Hvidovre Hospital. Eventual surplus will be used for remuneration of the study nurse affiliated to this trial. If not relevant, scholarships will be repaid to donors.

Trial participants will be reimbursed for their transportation expenses, after State rates and will be offered remuneration for additional time consumption, Dkr 1500,- in relation to this trial.

## Legal assistance

Legal advice for contracts and agreements is provided by Centre for Innovation and Research, The Capital Region of Denmark c/o Cobis, Ole Maaløes vej 3, DK-2200 Copenhagen N.

# Publication

The trial will be registered in the databases [www.clinicaltrials.gov](http://www.clinicaltrials.gov/) and [www.clinicaltrialsregister.eu](http://www.clinicaltrialsregister.eu/).

The study is expected to lead to more than one publication. The results will independent of outcome be sought published in leading journals within medicine (such as Lancet) or in the leading journals within the area of gastroenterology and hepatology (i.a. Gastroenterology or Hepatology).

Principle investigator Nina Kimer will be primary or corresponding author on major publications regarding this trial. Flemming Bendtsen, Søren Møller and Aleksander Krag, who outlined the study design, will be primary, secondary or final authors on publications regarding this study. Participating investigators will be co-authors according to their work and involvement in the trial.

# Implications

If intestinal decontamination with rifaximin alleviates the mechanism leading to decompensation and complications it may potentially have a clinical impact in advanced cirrhosis far beyond preventing recurrent hepatic encephalopathy. The aspects are prevention of spontaneous bacterial peritonitis in patients at risk of this disease, and potentially prophylaxis for patients with biochemical signs of bacterial translocation.

# List of appendices

Appendix 1: Protocol resume in Danish (for Science Ethics Committee)

Appendix 2: Participant information in Danish

Appendix 3: Written consent form in Danish

Appendix 4: Proxy Statement in Danish

Appendix 5: Guidelines for oral participant information in Danish (for Science Ethics

Committee)

Appendix 6: Investigational programme in English

Appendix 7: Contract between Sponsor/investigator and Norgine Denmark in English

Appendix 8: Sponsor/investigator agreement

Appendix 9: Contract between Sponsor/investigator and trial facilities in Danish

Appendix 10: Investigators Brochure, Rifaximin (for Danish Health and Medicines Authority)

Appendix 11: Investigational Medicinal Product Dossier, Rifaximin (for Danish Health and

Medicines Authority)

Appendix 12: Notification of producer (Orientering af fremstiller, for Danish Health and Medicines Authority)

Appendix 13: Example of medicine label (Etiket eksempel, for Danish Health and Medicines Authority)
